# The Use of Global Positioning and Accelerometer Systems in Age-Grade and Senior Rugby Union: A Systematic Review

**DOI:** 10.1186/s40798-021-00305-x

**Published:** 2021-02-22

**Authors:** Lee A. Bridgeman, Nicholas D. Gill

**Affiliations:** 1 Faculty of Sport, Health & Social Sciences,, Solent University, East Park Terrace, Southampton, SO14 0YN UK; 2 New Zealand Rugby Union, Wellington, New Zealand; 3 Adams Centre for High Performance, Faculty of Health, Sport and Human Performance, University of Waikato, Tauranga, New Zealand

**Keywords:** GPS, Distance, High-speed running, Collisions, Impacts, Peak periods

## Abstract

**Background:**

Global positioning systems (GPS) imbedded with accelerometer systems (AS) are used in rugby union (RU) to collect information on absolute and relative distances, distances in different speed zones, high-speed running (HSR) distances, repeated high-intensity efforts (RHIE) and collisions and impacts. This information can be used to monitor match play which can then be used to plan training sessions. The objective of this review was to conduct a systematic review of studies which have reported the use of GPS and AS.

**Methods:**

A systematic review of the use of GPS and AS in both age-grade and senior rugby was conducted. The authors systematically searched electronic databases from January 2010 until March 2020. Keywords included rugby union, GPS, global position* and microtechnology.

**Results:**

A total of 51 studies met the eligibility criteria and were included in this review. There was a total of 34 studies utilising GPS and AS in senior RU players (mean ± SD; age 26.2 ± 1.9 years; height 185.7 ± 2.6 cm; mass 101.3 ± 4.2 kg) and 17 studies in age-grade RU players (mean ± SD; age 17.6 ± 1.5 years; height 182.1 ± 3.3 cm; mass 87.1 ± 8.6 kg). The results of this review highlighted that there are differences between backs and forwards and within these positions in these groups during both match play and training sessions. The backs covered greater total absolute, relative and HSR distance compared to forwards. Forwards are involved in more collisions and impacts than backs. When investigating the most intense periods of match play, studies in this review highlighted that the demands during these periods outweigh the average demands of the game. It was proposed that a rolling average over different time epochs is the best way to assess this and ensure that the most intense periods of play are assessed and monitored.

**Conclusions:**

The information highlighted in this review can be used to help coaches assess performances in match play, allow them to plan appropriate training sessions and monitor training load.

**Supplementary Information:**

The online version contains supplementary material available at 10.1186/s40798-021-00305-x.

## Key Points

- Backs covered greater distances and at higher intensities than forwards in rugby union match play. There are also differences in the distances covered between playing positions (e.g. back row vs front row players) and between different levels of competition.
- The most intense periods of match play should be assessed so that the players are adequately prepared to perform during these periods.
- Game demands are different across every position, and the variation in metrics is likely influenced by many factors, including skill level, competition, game plan and environmental factors such as the weather.
- Individualised training and monitoring will allow players to be managed correctly in order for them to perform optimally during competition and stay healthy and injury-free.

## Introduction

Rugby Union (RU) has been classified as an intermittent high-intensity sport which involves maximum strength and power outputs, static efforts, collisions and impacts and high-speed running (HSR) interspersed with low-intensity efforts and rest periods [1–4]. The widespread use of microtechnology such as global positioning systems (GPS) and accelerometer systems (AS) [5] has allowed sports scientists and coaches to assess what happens during both training and matches [6–8] with the most common metrics in the English Premiership reported to be distance in speed zones followed by HSR and total distance covered [5].

Typically, players cover distances of between 5000 and 7000 m during matches with backs covering greater HSR distances while forwards are involved in more impacts [9], collisions and static work (e.g. mauls and scrums) [4, 10, 11]. In addition to HSR distance, players may also be involved in repeated high-intensity efforts (RHIE) (≥ 3 consecutive high-speed efforts or impacts occurring within 21 s) with an increase in capacity to do so reported to result in enhanced performance [12]. As well as allowing for the assessment of the average demands of RU match-play GPS and AS also allow for the assessment of maximal mean demands over specific periods of play. These periods of play have been reported to have far greater intensities than are evident when assessing average game demands and, therefore, knowledge of these periods may be useful [3].

Understanding the physical load players experience during match play through GPS and AS analysis may allow training sessions to be designed that replicate or indeed even exceed match-play demands in order to enhance performance [8, 13, 14]. It has also been suggested that this information could be used to identify players with the potential to progress to higher-level competition, understand the differences between different age-grade competitions and also to analyse the differences between competitions (i.e. Six Nations vs the Rugby Championship) [14]. In addition, the use of this data could be used to reduce the risk of injury through the monitoring of players’ training and match loads and identifying a player’s readiness to return to play post injury [5, 13].

Of concern to researchers and coaches alike is the reliability and validity of GPS units. A review into the use of GPS in team sports by Cummins et al. [8] concluded that GPS units have an acceptable level validity and reliability at low speeds and over longer distances. It should be noted though that the reliability of GPS units has been reported to be reduced at high speeds [15–18]. However, it was proposed that as long as these issues are taken into account when interpreting GPS data, the use of GPS devices to monitor and assess physical performance is warranted [8]. In addition to the locomotor data provided by GPS units, it has also been suggested that the use of integrated triaxial AS could allow for the measurement of impacts and collisions [8]. Accelerometers have been found to be reliable within and between devices in a laboratory setting and between devices in the field [19]. However, the measurement of impacts and collisions in RU may be limited by the ability of accelerometers to differentiate between types of impacts [8]. Therefore, the routine use of AS data may warrant further investigation before coaches feel comfortable utilising these metrics [5].

To the authors’ knowledge, the last review that investigated the use of GPS as part of a more comprehensive review on field performance in RU was in 2015 [9]. Therefore, the aim of this study was to conduct a systematic review of the use of GPS and AS in RU in order to get a clear picture of what information they provide and how this information can be beneficial to players, support staff and coaches. The period from 2010 and 2020 was selected by the authors for this review as it included studies published in the last 10 years and covers the three most recent RU World Cup (2011, 2015 and 2019) cycles.

## Methods

### Design

**Fig. 1.**
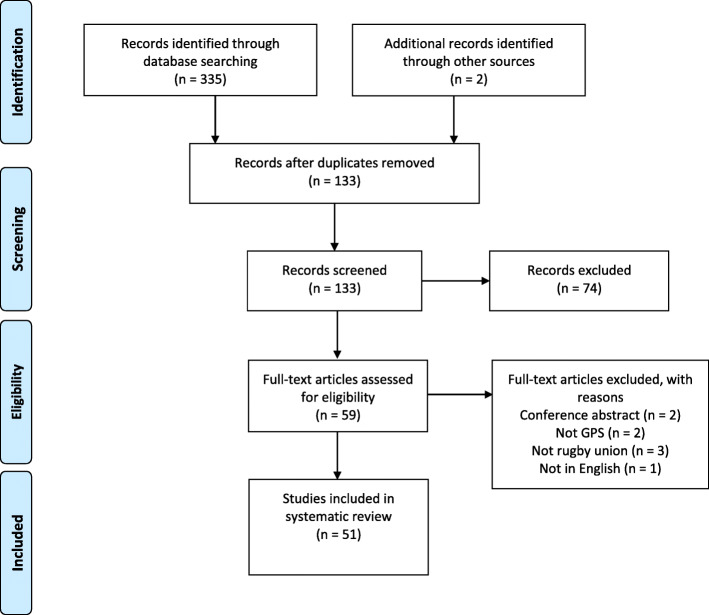
Results of study selection process

The review was conducted in line with the Preferred Reporting Items for Systematic Reviews and Meta-Analyses (PRISMA) guidelines [20]. One reviewer (LB) performed the initial database search for articles which investigated the use of GPS in senior and age-grade rugby union. The selected articles (titles and abstracts) were then reviewed by the other author (NG). Where any differences in opinion occurred, these were resolved through discussion. Searches were conducted using online databases PubMed, CINAHL, MEDLINE, Europe PMC and SPORTDiscus from January 2010 until March 2020. Keywords were grouped and searched within the article title, abstract and keywords using the Boolean operators ‘OR’ and ‘AND’. Combinations of the following terms were used as search terms: Rugby Union, GPS, Global Position* and Microtechnology. The search was limited to articles published in English and peer-reviewed journals. We also searched the references lists of each of the selected studies for any additional papers that should be included in this review. Full journal articles, investigating the use of GPS with male rugby union players and in the full fifteen a-side game, were selected for the systematic review (Fig. 1). Articles were excluded if they did not fulfil these criteria. In this review, studies that contained subjects who played professional club rugby or international rugby were categorised as senior rugby players. Those studies which included players who played at international under 20 (U20), university, academy or schoolboy/county (under 18 (U18) and 16 (U16)) level were categorised as age-grade rugby players.

### Data Extraction

Data relating to participants' characteristics, GPS device and GPS metrics; absolute total (m) and relative distance (m.min^−1^), HSR and very-high-speed running (VHSR) distance (m or m.min^−1^), RHIE efforts, collisions and impacts (frequency and magnitude as measured by AS) in matches, training and testing were extracted.

### Assessment of Methodological Quality

In line with previous reviews into the use of GPS in sport [8, 21], a modified assessment scale based on the work of Downs and Black [22], was utilised to evaluate the methodological quality of each study included in this review. Of Downs and Black's [22] original criteria, the 12 that were previously reported to be relevant to the study designs in this review were included [8, 21]. A meta-analysis was not performed as the data were unable to be pooled, due to the large variance in study designs (multiple different zones (HSR) and classifications (impacts and collisions).)

## Results

### Identification and Selection of Studies

The original database search identified 335 records. A total of 51 studies [3, 4, 6, 14, 18, 23–68] which met the eligibility criteria were included in this systematic review. An overview of the selection process is provided in Fig. 1.

### Review Characteristics

A summary of the methods and results of the studies which include the use of GPS in senior and age-grade male RU players is presented in Tables 1 and 2, respectively. There were a total of 1206 participants included in 34 studies [3, 4, 6, 18, 23, 26–34, 39–41, 43, 46–48, 50–53, 55, 58, 62–68] utilising GPS and AS in senior male RU players (mean ± SD; age 26.2 ± 1.9 years; height 185.7 ± 2.6 cm; mass: 101.3 ± 4.2 kg). Seventeen of these studies reported the number of data files extracted (*n* = 3151). There were 17 studies [14, 24, 25, 35–38, 42, 44, 45, 49, 54, 56, 57, 59–61] which utilised GPS and AS in age-grade male rugby union players (mean ± SD; age 17.6 ± 1.5 years; height 182.1 ± 3.3 cm; mass 87.1 ± 8.6 kg). Seven of these studies reported the number of data files extracted (*n* = 1476).

**Table 1:** Summary of senior rugby GPS studies

Study	Participants	Device details	Method	Results
Beard et al. [63]	188 rugby union players from the Pro12 and an international team.	Catapult Optimeye S5	Data were collected from GPS tracking devices (10 Hz) over the course of one Pro12 season and one international season. Total distance (m), relative distance (m.min^−1^), high-speed running (m.min^−1^) and max velocity was recorded for 6 positional groups and separated into club vs. international-level players.	Significant differences were found for repeated high-intensity locomotor efforts between club and international players in all position groups. Significantly greater total distance and relative distance was reported in international compared to club players for the outside back position.
Cahill [28]	120 professional rugby union players from the English Premiership.	GPSports SPI Pro	Data were collected from GPS tracking devices (5 Hz) during the 2010/11 competition. 8 professional clubs took part in the study. Total distance (m), relative distance (m.min^−1^), maximum speed (km h^−1^), average speed (km h^−1^) and total distance at different percentages of max velocity were recorded.	Results showed that the matches were played at a relatively slow pace with little distance covered in sprinting by both backs (50 ± 76 m) or forwards (27 ± 64 m). Backs covered greater absolute and relative distances compared to forwards (*p* < 0.05). Scrum halves covered the most distance during matches (7098 ± 778 m) and front row forwards the least (5158 ± 200 m).
Campbell et al. [52]	32 club rugby union players.	GPSports SPI HPU	Data were collected from GPS tracking devices (15 Hz) during a 19-week in competition period (training and matches).	Greater total distance (m), low-intensity activity, maximal speed and metres per minute were recorded during matches compared to training in all positions (*p* < 0.02).
Chambers et al. [64]	30 elite forwards.	Catapult Optimeye S5	Data were collected from GPS tracking devices (10 Hz) during both matches and training sessions. This allowed for the development of an algorithm to detect scrum events.	Across all positions the algorithm showed good sensitivity and specificity for training and match play. The algorithm displayed greater accuracy for match play than training (93.6 vs 87.6 %).
Chambers et al. [65]	12 elite rugby union players.	Catapult Optimeye S5	Data were collected from GPS tracking devices (10 Hz) during match play. Ruck and tackle data were synchronised with video footage of the games. The authors then developed an algorithm to detect tackles and rucks.	The algorithm was able to detect rucks and tackle for all positions. However, it does not provide the impact forces of these events.
Coughlan et al. [23]	2 players (1 back and 1 forward) from an international team.	GPSports Team AMS	Data were collected from GPS tracking devices (5 Hz) during 1 international rugby match for 1 back and 1 forward.	Players completed an average of 6715 m and spent the majority of the match standing or walking interspersed with medium and high-intensity running activities. The back performed a higher number of sprints and reached a greater maximal speed. Body load data showed high levels of G force are sustained during tackling and scrummaging.
Cousins et al. [66]	89 professional rugby union players from the top two leagues in England.	STATSport Apex	Data were collected from two GPS tracking devices (5 and 10 Hz) over 2 seasons. Total distance (m) and high-speed running distance (m) were recorded.	Distance covered had a significant influence on time-loss incidence (*p* < 0.001). For every 100 m extra distance covered there was a 1% increase in time-loss incidence. High-speed running distance also had a significant influence on time-loss incidence. For each 100 m increase in high-speed running distance there was a 21% increase in time-loss incidence.
Cunningham et al. [3]	119 elite professional players from three different international performance squads.	STATSport Viper Pod	Data were collected from GPS tracking devices (10 Hz) over a 3-year period (Jan 2014–March 2017). Two types of sampling-epoch were utilised. Rolling (ROLL) and fixed (FIXED) length epochs. An example of the use of the ROLL method is 60 s rolling-epoch algorithm is calculated using the current, and 599 preceding samples. For the fixed time method epochs were located at samples 1–600, 601–1200, 1201–1800 and so on.	Using both methods as the epoch length increased values for intensity of running decreased. Movement demands were underestimated consistently by the FIXED method.
Delaney et al. [6]	67 players from two international rugby union teams.	GPSports SPI HPU	Data were collected from GPS tracking devices (15 Hz) across 33 international matches. A moving average was used to identify the peak relative distance, average acceleration/deceleration (AveAcc: m s^−2^) and average metabolic power (*P*_met_) for a range of durations (1–10 min).	Peak running intensity increased as the length of the length of the moving average increased. Likely small to moderate increases in relative distance and AvcAcc for outside backs, half backs and loose forwards compared to the tight 5 group across all moving average durations (ES = 0.27–1.00). Metabolic power demands were at least greater for outside backs and half backs when compared to the tight 5 (0.86–0.99). Half-backs demonstrated greatest relative distance and *P*_met_ outputs but were similar to outside backs and loose forwards in AveAcc demands.
Dubois et al. [4]	14 professional rugby union players from the French Top 14.	GPSports Team AMS	Data were collected from GPS tracking device (5 Hz) from 5 European Cup games. Total distances, high-speed running distance, peak speed, number of sprints, number of accelerations and number of decelerations were reported.	Back covered greater distances at high-speed than forwards (*p* < 0.01). Forwards covered greater distances in the moderate speed zone (*p* < 0.05) than backs. No sig. differences in high-metabolic power distance were found between backs and forwards.
Dubois et al. [43]	8 professional rugby union players (all backs) from the D2 Championship in France.	Catapult, Minimaxx S4	Data were collected from GPS tracking devices (10 Hz) during training session over the course of the season. Total distance (m) and distance at moderate to high-speed (> 13 km h^−1^) were recorded.	Total distance covered per week was 19316 ± 2923 m and distance performed at moderate to high-speed was 3996 ± 701 m.
Grainger et al. [53]	38 professional rugby union players from the English Premiership.	STATSport Viper Pod	Data were collected from GPS tracking devices (10 Hz) over a 9-month in-season period. Both locomotor and collision data were reported.	No difference in the number of impacts > 9.01 G were observed between forwards and backs (229 ± 160 vs 226 ± 151). However, forwards had a greater absolute (*p* = 0.03) and relative (*p* = 0.003) number of impacts over 13 G. Full backs experienced the greatest frequency of absolute impacts > 9.01 G. and hookers experienced the greatest frequency of relative impacts > 9.01 G.
Jones et al. [30]	36 professional rugby union players.	Catapult, Minimaxx S4	Data were collected from GPS tracking devices (10 Hz) following 4 European Cup group matches during the 2012–2013 season. Both locomotor and collision data were reported.	Backs covered significantly greater total distance (m) compared to forwards (5959 ± 1013 vs 4906 ± 902, *p* < 0.01), greater distance per minute (67.8 ± 8.2 vs 60.4 ± 7.8, *p* < 0.01), performed a greater number of sprints (18 ± 6 vs 7 ± 6, *p* < 0.001), covered more distance (m) at high-speed (509 ± 150 vs 231 ± 167, *p* < 0.001) and covered more sprint distance (m) than forwards (333 ± 122 vs 121 ± 112, *p* < 0.001). However, forwards had a greater total number of contacts compared to backs (31 ± 14 vs 16 ± 7, *p* < 0.001).
Jones et al. [31]	33 professional rugby union players from a Pro 12 team.	Catapult, Minimaxx S4	Data were collected from GPS tracking devices (10 Hz) from 6 European Cup games and 7 Pro 12 games. Distances, velocities, accelerations, exertion index, player load, contacts, sprinting and repeated high-intensity efforts were reported.	Inside and outside back have the greatest high-speed running demands. Repeated high-intensity efforts and contact demands are greater in the loose forwards.
Lindsay et al. [32]	37 professional rugby union players from a Super Rugby squad.	Catapult, Minimaxx S4	Data were collected from GPS tracking devices (10 Hz) over 5 home games. Total distance (m) and distance covered in the following speed bands > 7 km h^−1^, 16 km h^−1^, > 20 km h^−1^ and > 25 km h^−1^was recorded.	Backs covered more metres per minute than forwards. Inside and outside backs covered a similar distance that was more than all the forward positions (*p* < 0.05). Backs covered significantly more distance per minute than forwards above 16, 20, 25 km h^−1^ (*p* < 0.01). Loose forwards covered more distance than locks and front rowers above 16, 20, 25 km h^−1^ (*p* < 0.01). Inside backs and outside backs covered more distance per min than all forward positions (*p* < 0.001).
MacLeod et al. [55]	37 professional rugby union players from the Pro12 competition.	STATSport Viper Pod	Data were collected from GPS tracking devices (10 Hz) from same team over 11 competitive matches. Collisions were automatically recorded using the GPS units.	Collision loads were significantly greater during dominant compared with neutral and passive collisions, tackles and carries (*p* < 0.001). Overall forwards reported a greater number and frequency of collisions but lower loads per collision and velocities at the point of collision compared to backs.
McLaren et al. [18]	28 professional rugby union players from the English Championship.	Catapult, Minimaxx S4	Data were collected from GPS tracking devices (10 Hz) from same team over 15 competitive matches during the 2012/13 season. Total distance (m), low speed running (0–14.9 km h^−1^), high-speed running (15.0–19.9 km h^−1^), and very-high-speed running (20.0–36.0 km h^−1^), PlayerLoad and PlayerLoad slow were reported.	Large between match variation (within-player) for high-speed and very-high-speed running and repeated high-intensity efforts for backs and forwards. PlayerLoad and PlayerLoad slow were reported to be more stable.
Owen et al. [33]	33 professional rugby union players from a Super Rugby squad.	GPSports SPI HPU	Data were collected from GPS tracking devices (15 Hz) by player position group over the first half of match play from 14 Super Rugby matches. Accelerations and decelerations, impacts, and aggregated body demands were reported.	Forwards had more high-intensity impacts (*d* = 0.44) and greater aggregated body demands (*d* = 0.26), while backs had more moderate (*d* = 0.55) and heavy accelerations (*d* = 0.76) and more moderate (*d* = 0.23) and heavy decelerations (*d* = 0.54).
Pollard et al. [58]	22 players from an international rugby team.	STATSport Viper Pod	Data were collected from GPS tracking devices (10 Hz). An Opta sportscode timeline was used in conjunction with GPS to split data into ball in play (BiP) times. Metres per min, high-metabolic load per min (HML), accelerations per min (Acc) high-speed running per min and collisions per min. Coll were expressed relative to BiP periods over the whole match.	Whole match metrics were sig lower than all BiP metrics (*p* < 0.001). Mean and max BiP HML (*p* < 0.01) and HSR (*p* < 0.05) were sig. higher for backs. Collisions were sig. higher for forwards (*p* < 0.01). In plays lasting 61 s or longer, max BiP m.min^−1^ were higher for backs. Max BiP m.min^−1^, HML, HSR and Coll were all time dependent. Movement metrics and collisions differ as length of play continues.
Reardon et al. [34]	36 professional rugby union players from a Pro 12 team.	Catapult Optimeye S5	Data were collected from GPS tracking devices (10 Hz). Total distance and total distance relative to playing time were calculated. Maximum velocity (Vmax) was calculated from all match and training data during the season to allow for the calculation of individual speed thresholds.	When comparing absolute to individualised HSR thresholds, there was a significant underestimation for forwards HSR distance (*p* < 0.001), HSR% (*p* < 0.001) and HSR efforts (*p* < 0.001). In contrast there was a sig. overestimation of the HSR metrics for backs with the use of an absolute threshold (*p* < 0.001 for all metrics).
Reardon et al. [47]	39 professional rugby union players from a Pro12 team.	Catapult Optimeye S5	Data were collected from GPS tracking devices (10 Hz) over 6 European Rugby Championship games and 11 games in the Pro 12.	Worst-case scenario (WCS) periods are played at a far higher pace than previously reported average game demands. Within WCS periods backs covered greater total distance than forwards (318 m vs 289 m), carried out more high-speed running (11.1 m.min^−1^ vs 5.5 m.min^−1^ and achieved the highest MaxVel values (6.84 m sec^−1^). Tight five and back row forwards had sig. more collisions than inside and outside backs (0.73 and 0.89 collisions m.min^−1^ vs 0.28 and 0.41 collisions m.min^−1^ respectively.
Reardon et al. [46]	36 professional rugby union players from a Guinness Pro 12 team.	Catapult Optimeye S5	Data were collected from GPS tracking devices (10 Hz) to monitor collision counts during match play. Collision thresholds were set between 2 and 5.5 g in 8 increments of 0.5 g. The upper threshold for all bands was 15 g.	Collision may be over or underestimated via GPS compared to expert video analysis. The use of 0.5 g increments of force did not provide a reliable tool for coding collisions.
Reid et al. [29]	8 professional rugby union players from a Magners League team.	GPSports SPI Pro	Data were collected from GPS tracking devices (5 Hz) during one league. Total distance (m), relative distance (m.min^−1^), time and distance in different speed zones and frequency of entry into each speed zone was recorded.	The backs covered a greater total distance than forwards, with the scrum half completing the most (7183.7 m) and the loose head prop the least (6206.2 m). The winger had the highest peak speed (31.1 km h^−1^) and most entries into the maximal speed zone (17). Backs spent less time and covered less distance walking than forwards.
Roe et al. [48]	9 professional rugby union players.	Catapult Optimeye S5	Players completed 3 maximal 40 m sprints with their maximum velocity assessed via timing gates, radar and a GPS tracking device (10 Hz).	The results of this study indicate that when compared with radar GPS was able to provide a valid measure of 40 m maximum velocity.
Suarez-Arrones et al. [26]	9 international rugby union players.	GPSports SPI Elite	Data were collected from GPS tracking devices (1 Hz) in forwards and backs during 3 competitive games. The frequency and duration of locomotor efforts were evaluated using distance covered in 6 zones.	Backs covered significantly greater total distance than forwards (6162 ± 313 m vs 5853 ± 205 m, *p* < 0.001). The forwards average speed during the games was 4.3 km h^−1^ and the backs 4.7 km·h^-1^.
Swaby et al. [39]	14 professional rugby union players from an English Premiership team.	STATSport Viper Pod	Data were collected from GPS tracking devices (10 Hz) during the first 6 matches of a season. Total distance (m) was the metric of interest.	No significant differences were observed on total distance between games. Greater distances were covered by backs during a game compared to forwards (6544 ± 573 m vs 4872 ± 857 m, *p* = 0.001). Maximum aerobic speed (MAS) performance showed a strong relationship with distance covered during match play (*r* = 0.746, *p* < 0.001).
Tee et al. [40]	53 professional rugby union players from a South African rugby team.	GPSports SPI Pro	Data were collected from GPS tracking devices (5 Hz) over 96 training sessions and 24 matches. GPS data were used to compare traditional rugby training activities (endurance, high-intensity interval, game-based and skills training) compared to match play. Movement patterns were measured as relative distance, distance walking, jogging, striding and sprinting and sprint and acceleration frequency	High-intensity interval training was the most similar to match play. Game based training failed to meet match intensity in all positions (ES = medium to large).
Tee et al [68]	19 professional rugby union players from a South African rugby team.	GPSports SPI Pro	Data were collected from GPS tracking devices (5 Hz) over 24 matches over the 2013 season. Movement patterns were measured as relative distance, distance walking, jogging, striding and sprinting and sprint and acceleration frequency. An inbuilt triaxial accelerometer (sampling at 100 Hz) measured total impacts > 5G and > 8G.	No difference between forwards and backs in relative distance covered (m.min^−1^). Backs covered more distance than forwards in high-intensity (striding and sprinting) speed zones. There were no differences in impact variables between forwards and backs.
Tee et al. [50]	19 professional rugby union players from a South African rugby team.	GPSports SPI Pro	Data were collected from GPS tracking devices (5 Hz) over a first-class professional season. Total relative distance (m.min^−1^), maximum speed, sprint frequency and acceleration frequency were reported.	Total relative distance (m.min^−1^) was decreased in the 2nd half for both forwards and backs (ES = *very likely* large). A larger reduction in high-intensity running distance in the 2nd half was observed in forwards.
Tee et al. [67]	19 professional rugby union players from a South African rugby team.	GPSports SPI Pro	Data were collected from GPS tracking devices (10 Hz) from 23 matches over the 2013 rugby season to assess pacing characteristics of whole or part-game players.	For forwards finishers who entered the game had significantly higher high-speed running distance (m) and acceleration frequency compared to whole game players. In the backs players who started but were later substituted displayed greater high-speed running distances compared to while game players (not statistically significant). Forwards were reported to show “slow positive” pacing strategies while backs had a “flat: pacing” strategy. Forward were reported to have greater decrements in performance as the match goes on.
Tierney et al. [51]	43 professional rugby union players from a Pro 12 team.	Catapult Optimeye S5	Data were collected from GPS tracking devices (10 Hz) over 11 European Rugby Championship and 11 Pro 12 games. Running intensity was calculated for total distance, running distance, high-speed running and very-high-speed running. The study also investigated attacking entries into the oppositions 22.	Forwards achieved greater high-speed running in successful (3.6 m.min^−1^) compared to unsuccessful (1.8 m.min^−1^) attacking 22 entries.
Vaz et al. [27]	40 rugby union players (20 experienced and 20 novice).	GPSports SPI Pro	Data were collected from GPS tracking devices (5 Hz) during eight 6 vs 6 matches over a 4-week period. Locomotor characteristics and impacts were recorded during these sessions.	Results showed no significant differences between experience and novice players.
Vaz et al. [41]	14 professional rugby union players.	GPSports SPI Pro	Data were collected from GPS tracking devices (5 Hz) during small-sided games during an in-season competition period. Four sessions were assessed during this study (1 vs 1, 2 vs 1, small-sided match 7 vs 7 and a match 7 vs 7). Speed zones, impacts, and relative distance (m.min^−1^) were recorded.	Different small-sided game set-ups resulted in different levels of physical performance.
Weaving et al. [62]	21 professional rugby union players.	Catapult, Minimaxx S4	Data were collected from GPS tracking devices (10 Hz) during training sessions over an entire season. Total distance (m), high-speed distance and PlayerLoad were calculated.	Mean total distance during training sessions was 3096 ± 675 m, high-speed distance was 127 ± 202 m and PlayerLoad was 292 ± 87 AU. For an individual total distance and PlayerLoad responded similarly to session RPE across training sessions. However, high-speed running provides unique information on the load.

**Table 2:** Summary of age-grade rugby GPS studies

Study	Participants	Device details	Method	Results
Carling et al. [42]	63 rugby union U20 international players from two teams.	STATSport Viper Pod	Data were collected from GPS tracking devices (10 Hz) during an U20 tournament. Players played 5 matches over 19 days.	Total and peak 5-min high-metabolic load distances were *likely-to-very likely* moderately higher in the final match compared to matches 1 and 2 in back and forward players.
Cunningham et al. [35]	40 rugby union U20 international players.	STATSport Viper Pod	Data were collected from GPS tracking devices (10 Hz) over 15 international tournament matches. Data on distances, velocities, accelerations, decelerations, high-metabolic load (HML) distance and efforts, and number of sprints were collected.	Analysis revealed sig. differences between forwards and backs. Backs scored higher on all variables measured with the exception of number of moderate accelerations (no sig differences).
Cunningham et al. [36]	43 rugby union U20 international players and 27 elite professional senior players from an international performance squad.	STATSport Viper Pod	Data were collected from a GPS tracking device (10 Hz) over 15 (U20) and 8 (senior) international tournament matches, Distance relative to playing time, HSR, number of sprints relative to playing time, mod, high and severe intensity accelerations and decelerations, high-metabolic load distance (HML) and high-metabolic load efforts were calculated.	Sig. differences between U20 and senior teams in both the forwards and backs. In the forwards seniors covered greater HML distance (*p* = 0.01) and severe decelerations (*p* = 0.05) in comparison to the U20s. However, they performed less relative HSR (*p* < 0.01), high accelerations (*p* < 0.01) and sprints·min^−1^ (*p* < 0.01). Senior backs covered a greater relative distance greater HML distance, HML efforts and heavy decelerations (all *p* < 0.01). U20 backs performed more relative HSR and sprints·min^−1^ (all *p* < 0.01).
Flanagan et al. [44]	42 rugby union U20 international players across two teams.	STATSport Viper Pod	Data were collected from GPS tracking devices (10 Hz) during 10 matches at the 2015 World Rugby U20 Championship. Distance total (m), relative distance (m.min^−1^), high-speed running, number of sprints and number of accelerations were recorded.	Mean running volumes ranged from 3994 to 6209 m with mean relative distances ranging from 56 to 71 m.min^−1^. During a 5-min maximal intensity period mean relative distance ranged from 77 to 100 m.min^−1^ with 16.6–31.9% of the distance covered at high-metabolic load.
Hartwig et al. [24]	118 rugby union players aged between 14 and 18 years.	GPSports SPI10	Data were collected via GPS (1 Hz) and video tracking to compare and contrast players training sessions with “typical match” demands. The data were collected during 2 rugby field training sessions and during one competitive match per week between 2003 and 2008 from 10 different teams representing 3 level of junior rugby. Sprint data and total time spent in different movement categories were the variables of interest.	Compared with training matches resulted in more time spent jogging (14 vs 8%), striding (3.2 vs 1.3%) and sprinting (1.3 vs 0.1%) (*p* < 0.001). Players were also found to cover greater distances (4000 ± 500 vs 2710 ± 770 m and performed more sprints (21.8 vs 1) during games compared to training (*p* < 0.001).
Lacome et al. [54]	24 rugby union U20 international players.	Digital Simulation Sensoreverywhere V2	Data were collected over the course of the 2016 U20 World Championship with GPS tracking devices (16 Hz). Players were divided into a high and a low exposure group. Total distance (m) and high-speed distance (m) were measured during training and matches	High-speed running was similar between both groups across the tournament. In the high exposure group high-speed running changed across the 5 successive matches. There was a *very likely* moderate difference in cumulated total distance covered by the high exposure group compared to the low exposure group.
Phibbs et al. [45]	170 adolescent rugby union players (U16 and U18).	Catapult Optimeye S5	Data were collected from GPS tracking devices (10 Hz) was used to calculate mean session training loads from 10 teams across 3 playing standards (school, club and academy) over the course of 1 in- season training week.	Under 18 players covered the highest total distance (4176 ± 433 m), run the furthest at high speed (1270 ± 288 m) and had the highest PlayerLoad (424 ± 56 AU). School level players had the lowest session loads in both age categories. Training loads and intensities increased with age and playing standard.
Phibbs et al. [57]	20 adolescent academy rugby union players.	Catapult Optimeye S5	Data were collected from GPS tracking devices (10 Hz) for each subject over a 10-week in-season period. Total distance (m), LSA distance (m), HRS distance (m), VHSR distance (m) and PlayerLoad (AU) and PlayerLoadSlow (AU) was recorded.	Mean weekly training distance was 11628 ± 3445 m and PlayerLoad was 1124 ± 330 AU. Mean total distance (13063 ± 3933 m vs 10195 ± 2242 m) and Playerload (1246 ± 345 vs 1002 ± 279 AU) were both *likely greater* for backs compared to forwards (moderate effect size).
Phibbs et al. [56]	61 adolescent schoolboy and academy rugby union players.	Catapult Optimeye S5	Data were collected from GPS tracking devices (10 Hz) during training (15 training sessions) and competitive matches (8 matches). Total distance (m), relative distance (m.min^−1^), PlayerLoad, MSS, relative MSS, LSA distance (m), relative LSA (m.min^−1^), HRS distance (m), relative HSR distance (m.min^−1^), VHSR distance (m) and relative VSHR (m.min^−1^) was recorded.	For the schoolboy forwards group, total PL and LSA were both *likely* greater in matches than training. In the schoolboy backs group, total distance MSS, LSA, HSR and relative VHSR were all *likely* greater in matches than training. For the academy forwards group relative PL and relative LSA were both *likely* greater in matches than training. In the academy backs group however, training demands were similar to match demands.
Read et al. [14]	112 rugby union representative players (U16, U18, U20).	Catapult Optimeye S5	Data were collected from GPS tracking devices (10 Hz) from 2 matches from each team (6 matches in total). Relative distance, LSR m.min^−1^, HRS m.min^−1^, PL m.min^−1^ and PL_slow_ m.min^−1^ were reported.	Backs had a greater relative distance (except U16s) and a greater high-speed running distance per minute than forwards with these magnitudes becoming larger with age. PlayerLoad per minute and PlayerLoad slow per minute was greater for forwards than backs at all age groups.
Read et al. [37]	96 rugby union players (U16, U18 and university).	Catapult Optimeye S5	Data were collected from GPS tracking devices (10 Hz) during six matches. Distance total (m), relative distance (m.min^−1^), maximum sprint speed (MSS), and total walking, jogging, striding and sprinting distances were reported. PL.min^−1^ was also reported.	U16 total distance and striding was likely higher for forwards than backs, at U18 level there were no clear differences and at university level this relationship was reversed. In all age groups sprint distance was *likely* greater for backs than forwards. Forwards had greater physical demands than backs at all age groups. Player demands were similar for forwards across age groups, and greater for back as age increased.
Read et al. [61]	202 rugby union players across 7 regional academies in England.	Catapult Optimeye S5	Data were collected from GPS tracking devices (10 Hz) across 24 matches from the U18 annual competitive league fixtures across three consecutive seasons. All matches were 35 min per half. Instantaneous speed was used to calculate relative distance using 0.1 s rolling mean for different times durations (15 and 30 s and 1, 2, 2.5, 3, 4, 5 and 10 min).	Running intensities for consecutive durations decreased as time increased. Running intensity was lower in the forwards than backs during all durations (ES = − 0.74 ± 0.21 to − 1.19 ± 0.21). Running intensity for the second row and back row positions was greater than the front row player at all durations (− 0.58 ± 0.38 to − 1.18 ± 0.29). Running intensity for scrum halves was greater (0.46 ± 0.43 to 0.86 ± 0.39) than inside and outside backs for all durations apart from 15 and 30 s.
Read et al. [60]	59 rugby union academy players from England.	Catapult Optimeye S5	Data were collected from GPS tracking devices (10 Hz) over two seasons totalling 12 matches. PL (PL·min^−1^) and relative distance (m.min^−1^), were synchronised with the timings of attack, defence and ball out of play time for analysis.	Relative distance in attacking phases (112.2 vs 114.6 m.min^−1^) was similar between forwards and backs. But greater in forwards during defensive plays (114.5 vs 109.0 m.min^−1^) and greater in backs during ball out of play.
Read et al. [59]	66 rugby union U18 academy and schoolboy players.	Catapult Optimeye S5	Data were collected from GPS tracking devices (10 Hz) during matches (6 academy and 6 schoolboy matches). Maximum sprint speed (MSS), and total walking, jogging, striding and sprinting distances were reported. PL_slow_.min^−1^ was also reported.	Academy forwards and backs *almost* certainly and *very likely* covered greater total distance than the schoolboys. Academy backs were *very likely* to accumulate greater PL_slow_ and academy forwards a *likely* greater sprinting distance than the schoolboys in their respective positions. The MSS, total, walking and sprinting distances were *greater* in backs (*likely-almost* *certainly*), forwards accumulated greater PL_slow_ (*almost* *certainly*) and jogging distances (*very* *likely*).
Roe et al. [38]	14 rugby union academy players.	Catapult Optimeye S5	Markers of fatigue were calculated before and after a competitive academy match. Locomotor demands were collected from a GPS tracking device (10 Hz).	Players covered an average of 4691 ± 878 m during the match. The average relative distance covered was 74 ± 6 m.min^−1^. Of the total distance 1771 ± 436 m was covered walking/standing, 2215 ± 461 m jogging, 663 ± 238 m striding and 41 ± 40 m sprinting.
Roe et al. [49]	20 rugby union academy players.	Catapult Optimeye S5	External training load was assessed over a 2-week period using GPS tracking devices (10 Hz) during both contact and non-contact sessions. Metrics recorded were total distance (m), relative distance (m.min^−1^) and PlayerLoad slow.	Having no contact in the session *almost certainly* increased running intensity (19.9 ± 5%) and distance (27.5 ± 5.3%).
Venter et al. [25]	17 semi-professional rugby union U19 players.	GPSports SPI Pro	Data were collected from GPS tracking devices (10 Hz) over 5 games during the in-season period. Total distance (m), speed zones and impacts were recorded.	Players covered an average of 4469.9 ± 292.5 m during the games. Players spent 72.32 ± 4.77% of the game either standing or walking. Back row forwards had the highest total amount of impacts during the game (683.4 ± 295.0) while the inside backs had the highest amount of severe impacts over 10 g power game (12.16 ± 3.18)

### Methodological Quality

The scores for the assessment of quality ranged from 6 to 10 (mean ± SD = 9 ± 1) across the 12 items that were assessed (see electronic supplementary Table 1). The main issue is that not all studies provided exact *p* values.

### Total Absolute Distance Covered in Match Play

#### Senior Rugby

**Fig. 2.**
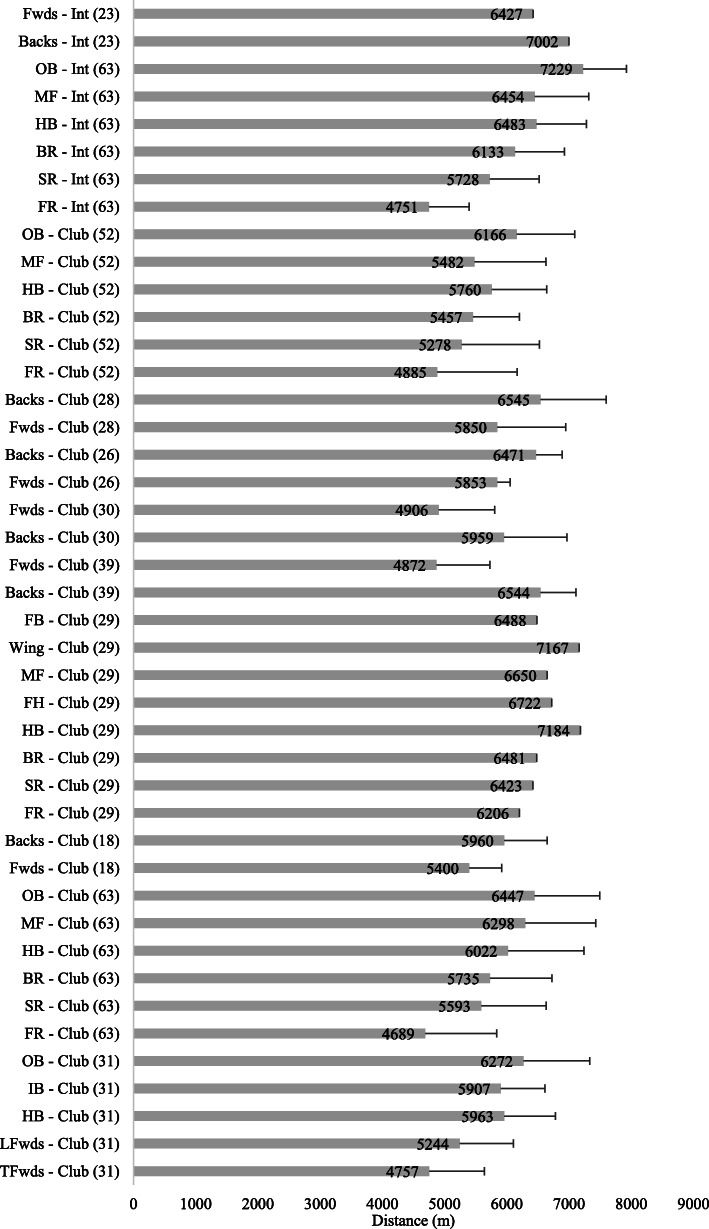
Total distance (m) covered in senior rugby match play (mean ± SD). TFwds tight forwards, LFwds loose forwards, HB half back/s, IB inside backs, OB outside backs, FR front row, SR second row, BR back row, MF midfield, Fwds forwards, FH fly half

A breakdown of the total absolute distances covered in senior match play by position is presented in Fig. 2. In the studies included in this review, international forwards covered a mean total distance of 5759 ± 731 m and backs 6792 ± 446 m [23, 63]. At professional club level, forwards covered 5476 ± 581 m and backs 6316 ± 446 m [18, 26, 28–31, 39, 52, 63]. When examining the studies individually, a number reported that senior backs covered significantly greater (*p* < 0.05) total absolute distance compared to forwards [4, 26, 28, 30]. In a study which specifically reported the differences between senior international and club players, Beard et al. [63] observed that international outside backs covered significantly more distance than club outside backs during match play (+ 10.8%, *p* < 0.05).

#### Age-Grade Rugby

**Fig. 3.**
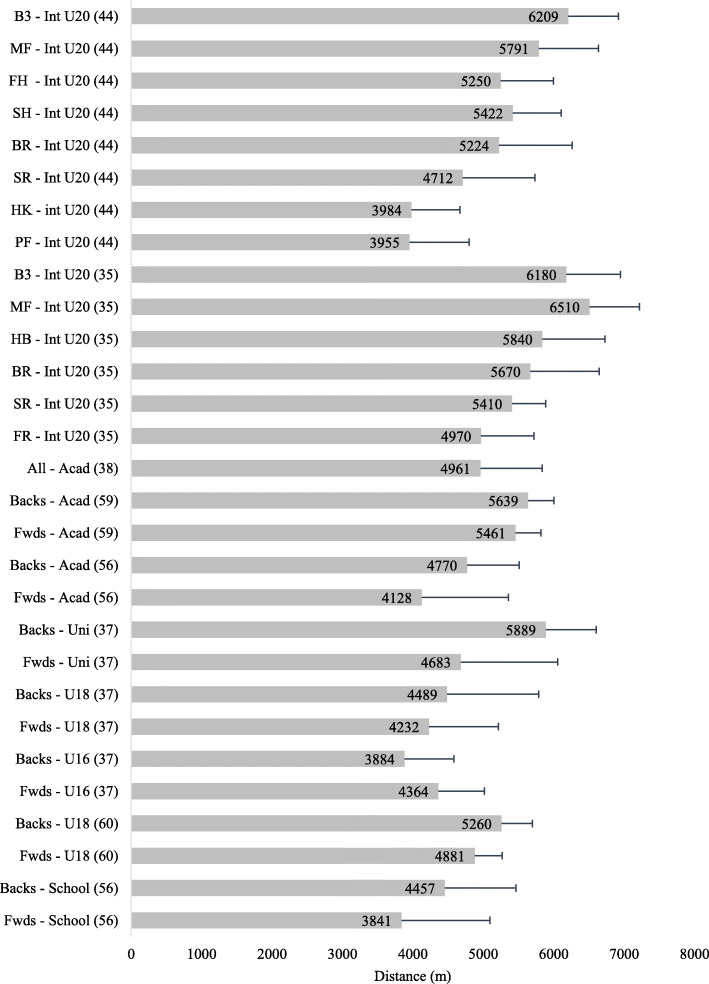
Total distance (m) covered in age-grade rugby match play (mean ± SD). TFwds tight forwards, LFwds loose forwards, HB half back/s, IB inside backs, OB outside backs, FR front row, SR second row, BR back row, MF midfield, Fwds forwards, FH fly half, B3 back three, HK hooker, SH scrum half

A breakdown of the total distances covered in age-grade matches by position is provided in Fig. 3. U20 international forwards covered 4846 ± 672 m and backs 5886 ± 449 m [35, 44]; academy forwards 4746 ± 1011 m and backs 5158 ± 679 m [38, 56, 59]; university forwards 4683 ± 1377 m and backs 5889 ± 719 m [37]; schoolboy forwards 4329 ± 429 m and backs 4522 ± 564 m [14, 37, 56, 59]. When examining the studies individually, it was observed that age-grade backs covered greater total absolute distance compared to forwards [35, 59]. In a study which investigated the physical demands of school and university match play, the authors reported that the forwards likely covered greater distance compared to the backs at U16 level. However, university forwards very likely covered less distance than the backs [37].

### Relative Distance Covered in Match Play

#### Senior Rugby

**Fig. 4.**
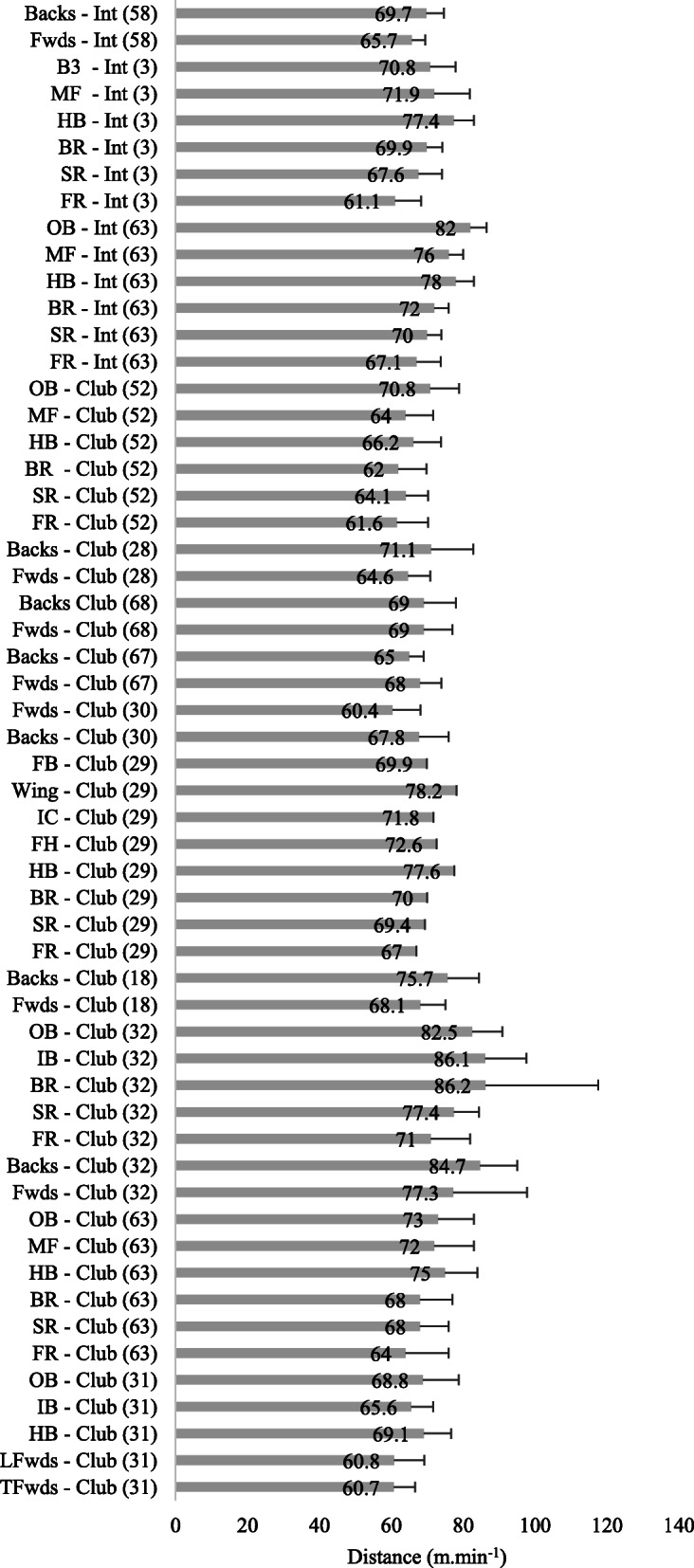
Relative distance (m.min^−1^) covered in senior rugby match play (mean ± SD). TFwds tight forwards, LFwds loose forwards, HB half back/s, IB inside backs, OB outside backs, FR front row, SR second row, BR back row, MF midfield, Fwds forwards, FH fly half

A breakdown of the relative distances covered in senior match play by position is provided in Fig. 4. International forwards covered 67.63 ± 3.57 m.min^−1^ and backs 75.11 ± 4.57 m.min^−1^ [23, 63]. At club level forwards covered 67.83 ± 6.51 m.min^−1^ and backs 71.99 ± 5.57 m.min^−1^ [18, 26, 28–31, 39, 52, 63, 68]. Further inspection of the individual studies revealed that senior backs covered significantly greater (*p* < 0.05) relative distance compared to forwards [28, 30, 32] during match play. As with total distance, Beard et al. [63] also observed that international outside backs covered significantly more relative distance than club outside backs (+ 12.3%, *p* < 0.05).

#### Age-Grade Rugby

**Fig. 5.**
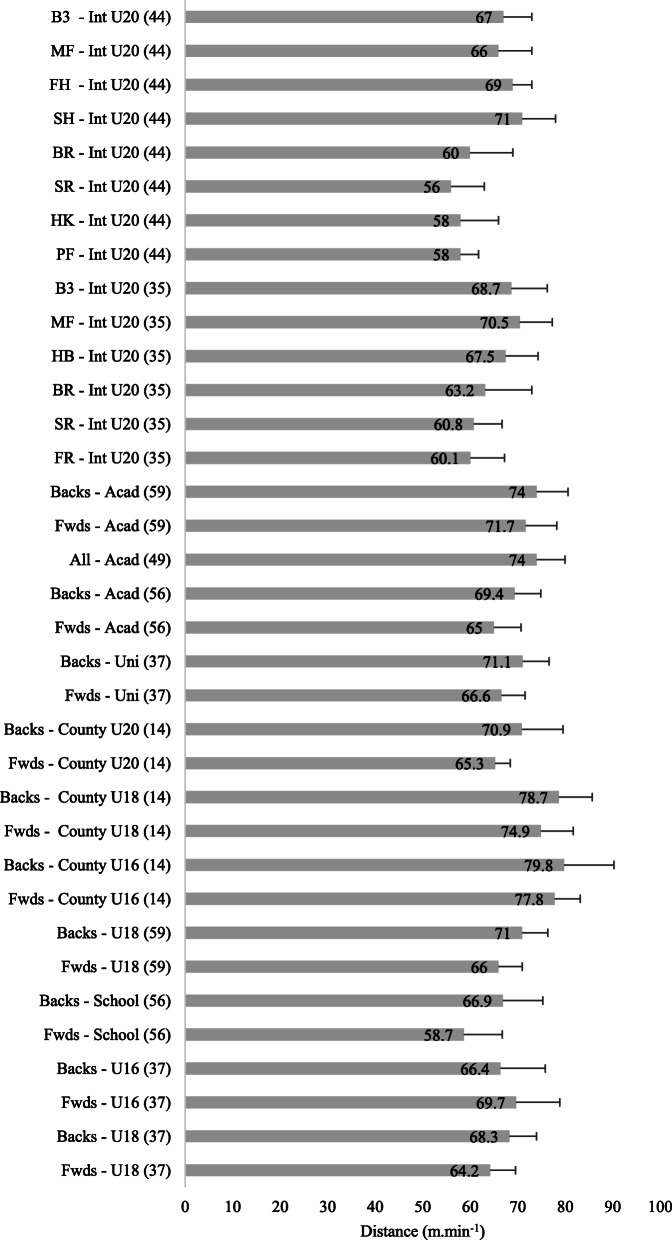
Relative distance (m.min^−1^) covered in age-grade rugby match play (mean ± SD). TFwds tight forwards, LFwds loose forwards, HB half back/s, IB inside backs, OB outside backs, FR front row, SR second row, BR back row, MF midfield, Fwds forwards, FH fly half, B3 back three, HK hooker, SH scrum half

A breakdown of the relative distances covered in age-grade matches by position is provided in Fig. 5. U20 international forwards covered 60.20 ± 3.23 m.min^−1^ and backs 69.09 ± 1.88 m.min^−1^ [35, 36, 44]; academy forwards 68.35 ± 4.74 m.min^−1^ and backs 71.70 ± 3.25 m.min^−1^ [56, 59]; university forwards 66.60 ± 5.00 m.min^−1^ and backs 71.10 ± 5.50 m.min^−1^ [37]; schoolboy forwards 68.55 ± 7.07 m.min^−1^ and backs 71.85 ± 5.96 m.min^−1^ [14, 37, 56, 59]. In a study which compared U20 and senior internationals, it was reported that the senior backs covered greater relative distance compared to age-grade backs (73.1 ± 8.1 m.min^−1^ vs 69.1 ± 7.6 m.min^−1^, *p* < 0.05) [36]. A further study reported that U18 and U20 backs covered a likely and very likely greater relative distance compared to forwards; however, this was not observed at the U16 level [14]. Another study observed that U16 forwards likely covered more relative distance than U18 forwards [37]. U18 forwards were also reported to cover likely less relative distance compared to the university forwards, and the same was true in the backs [37].

### High-Speed Running and Sprinting in Match Play

#### Senior Rugby

Due to the different threshold’s studies in this review used to identify HSR and sprints during senior match-play comparisons between individual studies is difficult. However, the HSR values reported in studies used in this review are displayed in Table 3. Four studies reported the HSR distances during international matches [23, 26, 58, 63] and ten during club matches [4, 18, 28, 30–32, 34, 52, 58, 67]. Pollard et al [58] reported that the backs completed more HSR than the forwards in international rugby (7.5 ± 1.9 m.min^−1^ vs 3.3 ± 1.5 m.min^−1^, *p* < 0.05). At club level, Dubois et al. [4] observed the backs also completed more HSR compared to the forwards (537.1 ± 127 m vs 397.2 ± 117.9 m, *p* = 0.0002). Similar values were reported by Jones et al. [30] who found that the backs also completed more HSR (509 ± 150 m vs 231 ± 167 m, *p* < 0.0001). Jones and colleagues [30] also reported moderate ES correlations between HSR distance and changes in creatine kinase (CK) concentrations post-match for the backs. In Super Rugby, backs were also reported to have completed more HSR compared to forwards [32].

**Table 3:** Summary of high-speed running (HSR) and sprinting in senior rugby

Study	Participants	Forwards	Backs
Beard et al. [63]	188 rugby union players from the Pro12 and an international team.	**Distance > 5.6 ms**^**−1**^ International front row—59 ± 54 m Club front row—49 ± 54 m International second row—161 ± 105 m Club second row—103 ± 69 m International back row—206 ± 110 m Club back row—168 ± 102 m **Distance > 7.5 ms**^**−1**^ International front row—0.4 ± 2.3 m Club front row—0.3 ± 2.0 m International second row—3.2 ± 7.6 m Club second row—2.1 ± 7.5 m International back row—12.1 ± 20.5 m Club back row—5.9 ± 13.1 m	**Distance > 5.6 ms**^**−1**^ International half back—338 ± 121 m Club half back—299 ± 123 m International centre—455 ± 160 m Club centre—376 ± 144 m International outside back—577 ± 204 m Club outside back—441 ± 143 m **Distance > 7.5 ms**^**−1**^ International half back—30.9 ± 31.0 m Club half back—24.8 ± 28.3 m International centre—34.3 ± 40.8 m Club centre—29.4 ± 30.0 m International outside back—101.7 ± 78.9 m Club outside back—61.4 ± 48.5 m
Cahill [28]	120 professional rugby union players from the English Premiership.	**51–80% Vmax** Median 860 m IQR 440 m **81–95% Vmax** Median 37 m IQR 64 m **96–100% Vmax** Median 0 m IQR 6 m	**51–80% Vmax** Median 822 m IQR 338 m **81–95% Vmax** Median 50 m IQR 76 m **96–100% Vmax** Median 0 m IQR 8 m
Campbell et al. [52]	32 club rugby union players.	**HSR > 5.5 ms**^**−1**^ Front row—78 ± 76 m Seconds row—159 ± 124 m Loose forwards –159 ± 124 m **Sprints** Front row—0.1 ± 0.07 m.min^−1^ Seconds row—0.1 ± 0.08 m.min^−1^ Loose forwards –0.2 ± 0.09 m.min^−1^	**HSR > 5.5 ms**^**−1**^ Half backs—244 ± 110 m Centres—308 ± 152 m Outside backs 400 ± 170 m **Sprints** Half backs—0.3 ± 0.09 m.min^−1^ Centres—0.3 ± 0.07 m.min^−1^ Outside backs 0.2 ± 0.09 m.min^−1^
Coughlan et al. [23]	2 players (1 back and 1 forward) from an international team.	**HSR 5–6.7 ms**^**−1**^ 5.6% **Max speed running > 6.7 ms**^**−1**^ 0.3%	**HSR 5–6.7 ms**^**−1**^ 7.5% **Max speed running > 6.7 ms**^**−1**^ 1.6%
Cunningham et al. [36]	43 rugby union U20 international players and 27 elite professional senior players from an international performance squad.	**HSR > 5.0 ms**^**−1**^ 3.1 ± 1.6 m.min^−1^ **Sprints** 0.11 ± 0.06 m.min^−1^	**HSR > 5.0 ms**^**−1**^ 7.2 ± 2.1 m.min^−1^ **Sprints** 0.25 ± 0.07 m.min^−1^
Dubios et al. [4]	14 professional rugby union players from the French Top 14.	**HSR > 4 5 ms**^**−1**^ 397.2 ± 117.9 m	**HSR > 4 5 ms**^**−1**^ 537.1 ± 127.2
Jones et al. [30]	36 professional rugby union players.	**HSR > 5 ms**^**−1**^ 231 ± 167 m **Sprinting > 5.6 ms**^**−1**^ 121 ± 112 m	**HSR > 5 ms**^**−1**^ 509 ± 150 m **Sprinting > 5.6 ms**^**−1**^ 333 ± 122 m
Jones et al. (3§)	33 professional rugby union players from a Pro 12 team.	**HSR 5–5.5 ms**^**−1**^ Tight forwards—81 ± 43 m Loose forwards—140 ± 63 m **Sprinting > 5.6 ms**^**−1**^ Tight forwards—64 ± 46 m Loose forwards—166 ± 116 m	**HSR 5–5.5 ms**^**−1**^ Half backs—155 ± 71 m Inside backs—209 ± 56 m Outside backs—174 ± 52 m **Sprinting > 5.6 ms**^**−1**^ Half backs—226 ± 112 m Inside backs—378 ± 149 m Outside backs—392 ± 135 m
Lindsay et al. [32]	37 professional rugby union players from a Super Rugby squad.	**HSR > 5.5 ms**^**−1**^ Front row—1.4 ± 1.1 m.min^−1^ Locks—1.3 ± 0.8 m.min^−1^ Loose forwards—5.3 ± 2.0 m.min^−1^ **Very HSR > 6.9 ms**^**−1**^ Front row—0.01 ± 0.01 m.min^−1^ Locks—0.05 ± 0.01 m.min^−1^ Loose forwards—0.61 ± 0.66 m.min^−1^	**HSR > 5.5 ms**^**−1**^ Inside backs—5.9 ± 2.6 m.min^−1^ Outside backs—7.1 ± 2.0 m.min^−1^ **Very HSR > 6.9 ms**^**−1**^ Inside backs—0.75 ± 0.73 m.min^−1^ Outside backs—2.14 ± 1.20 m.min^−1^
Mclaren et al. [18]	28 professional rugby union players from the English Championship.	**HSR 4.1–5.5 ms**^**−1**^ 650 ± 160 m **Very HSR > 5.5 ms**^**−1**^ 180 ± 110 m	**HSR 4.1–5.5 ms**^**−1**^ 770 ± 240 m **Very HSR > 5.5 ms**^**−1**^ 400 ± 130 m
Pollard et al. [58]	22 players from an international rugby team.	**HSR > 5 ms**^**−1**^ 3.3 ± 1.5 m.min^−1^	**HSR > 5 ms**^**−1**^ 7.8 ± 1.9 m.min^−1^
Reardon et al. [34]	36 professional rugby union players from a Pro 12 team.	**HSR absolute > 5 ms**^**−1**^ 269 ± 172.02 m **Individualised HSR** 354.72 ± 99.22 m	**HSR absolute > 5 ms**^**−1**^ 697.79 ± 198.11 m **Individualised HSR** 570.02 ± 171.14 m
Reid et al. [29]	8 professional rugby union players from a Magners League team.	**HSR 5.1–6.7 ms**^**−1**^ Loose head prop—260 m Lock—134.7 m Open side flanker—595.6 m **Max speed running > 6.7 ms**^**−1**^ Loose head prop—12.4 m Lock—0 m Open side flanker—84.2 m	**HSR 5.1–6.7 ms**^**−1**^ Scrum half—645.9 m Fly half—416.3 m Inside centre—550.9 m Wing—607.5 m Full back—472.2 m **Max speed running > 6.7 ms**^**−1**^ Scrum half—57.4 m Fly half—0 m Inside centre—46.5 m Wing—159.1 m Full back—77.6 m
Suarez-Arrones et al. [26]	9 international rugby union players.	**High intensity 5–5.5 ms**^**−1**^ Front row—75 ± 16 m Back row—190 ± 34 m **Sprinting > 5.5 ms**^**−1**^ Front row—86 ± 39 m Back row—232 ± 37 m	**High intensity 5–5.5 ms**^**−1**^ Centres—292 ± 91 m Scrums/out—252 ± 148 m **Sprinting > 5.5 ms**^**−1**^ Centres—633 ± 47 m Scrums/out—292 ± 44 m
Tee et al. [67]	19 professional rugby union players from a South African rugby team.	**HSR > 4 ms**^**−1**^ 10 ± 4 m.min^−1^	**HSR > 4 ms**^**−1**^ 12 ± 3 m.min^−1^
Tee et al. [68]	19 professional rugby union players from a South African rugby team.	**HSR > 4 ms**^**−1**^ 11 ± 5 m.min^−1^	**HSR > 4 ms**^**−1**^ 14 ± 4 m.min^−1^

In a study which compared individualised (HSR%) to absolute speed thresholds (HSR), it was reported that using absolute thresholds in forwards underestimated HSR distance (absolute = 269 ± 172.02 m vs individualised = 354.72 ± 99.22 m, *p* < 0.001), HSR% (absolute = 5.15 ± 3.18% vs individualised = 7.06 ± 2.48%, *p* < 0.001) and the number of HSR efforts (absolute = 18.81 ± 12.25 vs individualised = 24.78 ± 8.30, *p* < 0.001) compared to individual thresholds [34]. In backs, the opposite was reported to be true with absolute speed thresholds resulting in a significant overestimation of HSR distance (absolute = 697.79 ± 198.11 m vs individualised = 570.02 ± 171.14 m, *p* < 0.001), HSR% (absolute = 10.85 ± 2.82% vs individualised = 8.95 ± 2.76%, *p* < 0.001) and HSR efforts (absolute = 41.55 ± 1.25 vs individualised = 34.54 ± 9.24, *p* < 0.001) compared to individual thresholds [34].

#### Age-Grade Rugby

The match-play HSR values reported during age-grade match play are displayed in Table 4. Three studies reported the HSR distances covered during U20 internationals [35, 36, 44]. In a study which investigated the movement demands of U20 internationals versus senior internationals, it was reported that the age-grade players completed more relative HSR distance in both the backs and the forwards [36]. When broken down into positional groups, it was observed that the age-grade players covered greater relative HSR distance in the front row, second row and back three positions while the senior midfield covered more distance than the younger players [36]. A further study by Cunningham et al. [35] reported that in U20 internationals, both absolute and relative HSR distance was greater in backs than forwards (*p* < 0.05). When analysed by position, the authors reported that front row covered the least HSR distance compared to the back three who covered the most [35]. Flanagan et al. [44] compared individualised (> 67.5% Vmax) and absolute HSR (> 5.5 m s^−1^) thresholds in U20 international players. Similar to the work of Reardon at al [34]., the authors reported that in all forward positions, individualised HSR was greater than the absolute HSR. In the backs, it was reported that absolute HSR was greater than relative HSR for all positions except scrum half [44].

**Table 4:** Summary of high-speed running (HSR) and sprinting in age-grade rugby matches

Study	Participants	Forwards	Backs
Cunningham et al. [35]	40 rugby union U20 international players.	**HSR > 5.0 ms**^**−1**^ Overall mean—284.2 ± 134.9 m Front row—211.6 ± 112.7 m Second row—265.3 ± 94.2 m Back row—359.7 ± 142.7 m **Sprints** Overall mean—0.11 ± 0.05 m.min^−1^ Front row—0.09 ± 0.04 m.min^−1^ Second row—0.10 ± 0.03 m.min^−1^ Back row—0.14 ± 0.05 m.min^−1^	**HSR > 5.0 ms**^**−1**^ Overall mean 656.9 ± 182.7 m Half back—476.1 ± 204.1 m Midfield—661.7 ± 145.1 m Back three—728.4 ± 150.2 **Sprints** Overall mean—0.26 ± 0.07 m.min^−1^ Half back—0.18 ± 0.06 m.min^−1^ Midfield—0.27 ± 0.06 m.min^−1^ Back three—0.29 ± 0.06 m.min^−1^
Cunningham et al. [36]	43 rugby union U20 international players and 27 elite professional senior players from an international performance squad.	**HSR > 5.0 ms**^**−1**^ 3.2 ± 1.5 m.min^−1^ **Sprints** 0.11 ± 0.05 m.min^−1^	**HSR > 5.0 ms**^**−1**^ 7.3 ± 2.1 m.min^−1^ **Sprints** 0.26 ± 0.07 m.min^−1^
Flanagan et al. [44]	42 rugby union U20 international players across two teams.	**HSR absolute > 5.5 ms**^**−1**^ Prop—44 ± 42 m Hooker—88 ± 88 m Second row—55 ± 66 m Back row—153 ± 65 m **HSR Individual** Prop—100 ± 58 m Hooker—104 ± 60 m Second row—85 ± 76 m Back row—212 ± 99 m	**HSR absolute > 5.5 ms**^**−1**^ Scrum half—191 ± 80 m Outhalf—123 ± 29 m Centre—363 ± 120 m Back three—514 ± 153 m **HSR Individual** Scrum H = half—318 ± 300 m Outhalf—118 ± 31 m Centre—277 ± 159 m Back three—296 ± 99 m
Hartwig et al. [24]	118 rugby union players aged between 14 and 18 years.	**Striding 3.3–5.8 ms**^**−1**^ 3.6 ± 3.5% **Sprinting > 5.8 ms**^**−1**^ 0.9 ± 2.1%	**Striding 3.3–5.8 ms**^**−1**^ 3.1 ± 1.8% **Sprinting > 5.8 ms**^**−1**^ 1.3 ± 0.8%
Phibbs et al. [56]	61 adolescent schoolboy and academy rugby union players.	**HSR > 61% maximal sprint speed (m)** Schoolboy—138 ± 114 m Academy—220 ± 111 m **VHSR > 90% maximal sprint speed (m)** Schoolboy—0 ± 1 m Academy—5 ± 10 m	**HSR > 61% maximal sprint speed (m)** Schoolboy—359 ± 182 m Academy—280 ± 96 m **VHSR > 90% maximal sprint speed (m)** Schoolboy—19 ± 24 m Academy—15 ± 15 m
Read et al. [37]	96 rugby union players (U16, U18 and university).	**Striding 3.33–5.83 ms**^**−1**^ U16—993 ± 295 m U18—995 ± 370 m University—1112 ± 442 m **Sprinting > 5.83 ms**^**−1**^ U16—87 ± 86 m U18—94 ± 93 m University—64 ± 65 m	**Striding 3.33–5.83 ms**^**−1**^ U16—843 ± 342 m U18—1009 ± 444 m University—1460 ± 357 m **Sprinting > 5.83 ms**^**−1**^ U16—165 ± 101 m U18—319 ± 176 m University—353 ± 147 m
Roe et al. [38]	14 rugby union academy players.	**No positions stated in results for HSR** **Jogging 20–50% Vmax** 2215 ± 461 m **Striding 51–80% Vmax** 663 ± 238 m **Sprinting 81–95% Vmax** 41 ± 40 m	
Venter et al. [25]	17 semi-professional rugby union U19 players.	**Striding 50–79% Vmax** Front row—9.58 ± 4.59% Back row—6.04 ± 1.83% **Sprinting 80–95% Vmax** Front row—0.42 ± 0.22% Back row—0.42 ± 0.12% **Maximum sprint > 95% Vmax** Front row—0.06 ± 0.01% Back row—0.05 ± 0.02%	**Striding 50–79% Vmax** Inside backs—6.22 ± 3.67% Outside backs—2.84 ± 0.45% **Sprinting 80–95% Vmax** Inside backs—0.66 ± 0.26% Outside backs—1.05 ± 1.15% **Maximum sprint > 95% Vmax** Inside backs—0.06 ± 0.04% Outside backs—0.06 ± 0.04%

The remaining studies reported the HSR values during schoolboy, university, academy and age-grade semi-professional rugby [14, 24, 25, 37, 38, 56]. In a study which investigated the U16, U18 and university players, Read et al. [37] reported that the forwards covered less sprinting distance compared to the backs at all levels of match play. A further study reported that U16 forwards covered possibly and almost certainly less HSR compared to U18 and U20 forwards, respectively [14]. In the backs, it was reported that U16 players very likely completed less HSR compared to U18 backs and U18 backs almost certainly completed less HSR compared to U20 players [14].

## Discussion

The purpose of this review was to provide a summary of the current research in age-grade and senior RU utilising GPS and AS. The results of this review and their potential applications are discussed in the following sections.

### Running during Match Play

#### Total Distance, Relative Distance and High-Speed Running

The research in this review highlights that both senior and age-grade backs generally cover greater total [28, 32, 35], relative [14, 28] and HSR [4, 14, 32, 36] distance compared to the forwards. However, the current authors suggest that there is a need to ensure that the individual positions within these groups are assessed separately to ensure they are ready to perform on match day. An example of this is the findings that the tight forwards (front and second row) were similar in measures of total distance, HSR and sprints but significantly different to other playing positions possibly as a result of them being involved more at the breakdown and in set pieces [35]. Therefore, if the loose forwards were grouped with the tight forwards during all training drills and sessions, they could be underprepared come match day. This highlights that a one size fits all approach is not appropriate and a generic training approach may not enhance performance. Therefore, individual monitoring of players in all positions is advised.

The results of studies which have investigated the differences between playing standards may also be of interest to coaches and players as they provide information on what to expect as players transition towards higher levels of representative rugby. Cunningham et al. [36] investigated the differences in movement demands between elite U20 and senior international rugby union players. In this study, they reported significant differences between the teams; in the forwards, the senior players had more severe decelerations, but less relative HSR distance, moderate and high accelerations and sprints compared to the U20 players. In the backs, it was reported that the seniors covered a greater relative distance and did more intense decelerations. However, it was also found that the U20 backs completed more relative HSR distance and sprints. Of interest is the finding that the U20 front row performed more HSR, moderate and intense accelerations and all decelerations. These results, the authors propose, are the result of the senior players having more transient fatigue from being involved in more static exertions with higher loads than their younger counterparts [36]. Therefore, when considering the differences, it is suggested by the current authors that the GPS results should not be taken in isolation and instead the characteristics of the subjects (particularly increases in mass) need to be considered as these may have an impact on the running capabilities of players.

At schoolboy and academy level, Read et al. [59] reported that academy rugby players experienced greater match-play demands compared with their schoolboy counterparts. The authors suggested that as players can sometimes be expected to perform for both teams concurrently, it is vital that schoolboy players are exposed to the demands of academy rugby during training to adequately prepare them and aid their progression to senior rugby [59]. When assessing GPS and AS data, it is also essential that coaches recognise that all matches differ due to the team’s overall game plan for that match, a player’s role within the game plan, the level of competition, how the opposition play and environmental factors such as the weather. As a result, it is incumbent on those reporting GPS and AS data to ensure they fully understand the overall big picture and context of the match when interpreting data. Therefore, the authors suggest more research is required to establish how the context of a match can affect a player’s GPS and AS metrics.

In a study which investigated which aspects of match performance affected post-match CK responses, Jones and colleagues [30] reported moderate ES correlations between HSR distance and changes (CK) post-match for the backs. This they proposed was the result of high force, eccentric work that takes place when performing HSR [30]. As CK concentration is a marker for muscle damage, the amount of HSR a back does during match play should be taken into account by coaches planning recovery strategies and the following week’s training. The authors acknowledge that correlation does not equal causation and that more research is required to establish this relationship. However, based on experience, it is logical that the greater amount of HSR a player completes will have an impact on their recovery and subsequent ability to train optimally. However, as with all the metrics available to coaches, HSR should not be considered in isolation as although the backs do more HSR than forwards the forwards will be involved in more static exertions such as scrums and mauls. The effect of these static exertions also needs to be taken into account when planning post-match recovery and future sessions.

One issue with comparing studies which have utilised GPS in rugby union is the use of default or absolute speed zones in some studies and the use of relative or individualised speed zones in others [34]. Reardon et al. [34] propose that reporting running demands in relation to pre-determined speed zones is likely to result in over- or underestimation of the HSR requirements of players. In a study that investigated the application of individualised speed thresholds, Reardon et al. [34] reported the use of absolute compared to individualised HSR threshold resulted in a significant underestimation of HSR distance, HRS% and HRS efforts in forwards. The same authors also reported a significant overestimation of the same HSR metric for backs when utilising absolute thresholds [34]. In agreement with these findings, Flanagan et al. [44] reported that in all U20 international forward positions, individualised HSR was greater than the absolute HSR. In the backs, it was also reported that absolute HSR was greater than relative HSR for all positions except scrum half [44]. In a study which used individual thresholds for HSR which were assessed during match play, Cahill et al. [28] reported in contrast to other studies that the forwards actually sprinted more than the backs. However, as the maximum velocity used to determine HSR zones was assessed during match play, the current authors suggest that this resulted in the forwards’ having to achieve slower velocities for them to be recorded as a sprint and therefore inflated the forwards' sprint number. Therefore, should coaches wish to use individual thresholds, the method utilised to assess maximum velocity (match play maximum velocity vs maximum velocity achieved during training/testing sessions) needs to be considered as this will influence HSR outputs. When considering the use of absolute versus individual threshold Flanagan and colleagues [44] proposed that individual thresholds may be better to describe an individual player’s performance and manage that player’s load. These individual profiles allow coaches to establish what a normal match looks like for each individual and plan future training sessions around these observations. However, as rugby is a game of absolutes using absolute thresholds allows players to be assessed against others to see who potentially is ready to step and achieve higher honours and can assess a player’s absolute contribution during games [44]. Based on these findings, the current authors suggest it may be useful to use both types of thresholds to allow for a complete picture; however, it is acknowledged that this may be time consuming and not always practically possible.

#### Repeated High-Intensity Efforts

Beard and colleagues [63] investigated the differences between professional club players and international players. They reported that repeated high-intensity locomotor efforts (RHILE) (three or more accelerations and HSR with < 21 s of recovery) were significantly higher (*p <* 0.05) for international compared to club players in all position groups [63]. It was also reported that international outside backs covered significantly greater total distance at higher intensities (*p <* 0.05) compared with club players [63]. This led the authors to conclude that training methods that focus on repeated sprint and repeated high-intensity locomotive efforts should be prioritised for international players [63]. In addition, Beard et al. [63] sensibly suggested that as club players prepare to join up with their international teams, they should increase their efforts in club training to ensure they are prepared for the challenges of international rugby. This provides evidence of the need for good lines of communication and cooperation between both clubs and international teams to ensure players being considered for an international call up are adequately prepared to make the step up. A further study investigating repeated high-intensity efforts (RHIE; ≥ 3 consecutive high-speed efforts or impacts (tackle, scrum, ruck and maul) occurring within 21 s) observed that forwards completed 25.6 ± 5.7 efforts versus backs 28 ± 13 in club matches [18]. When considering differences in RHIE between different positions in senior club rugby, Jones et al. [31] reported that loose forwards completed more RHIE bouts compared to half backs and outside backs (*p* < 0.05). In summary, this information further highlights the need for bespoke training programmes which take into account the playing demands of different positions, the context in which the game is played, the level of competition and the opposition.

#### Fatigue During Match Play

When examining temporal differences utilising GPS, Jones et al. [31] reported that players showed significant reduction in repeated high-intensity effort bouts and contacts at the 50–60-, 60–70- and 70–80-min marks compared to 40–50 min. In addition, this study reported an increase in high-intensity, sprinting and high-speed meterage during the final 10 min of the match to values not statistically different to any other 10-min period [31]. The authors suggested that this was evidence that players may reduce the amount of low-intensity work they do towards the end of the match in order to still be able to perform high-intensity movements [31]. It should be noted however that there is no mention of the results of the matches from this study and the results may be indicative of close matches where the players, by necessity, needed to perform at high intensities during the final 10 min in order to chase a match or secure a win.

In a study which investigated the impact of fatigue on positional movements, Tee et al. [50] reported a ~ 10% decrease in distance covered per minute from the first half to the second half for both backs and forwards. There were, however, some differences reported between backs and forwards across the course of the matches [50]. Forwards were found to have a decrease in high-intensity running distance, sprint and acceleration frequency across halves, whereas no significant decrease was found for backs [50]. The authors concluded that the onset of fatigue occurs relatively early in forwards compared to backs with this suggested to be the result of the large number of collisions and static efforts that forwards are involved in compared to backs [10, 69]. Therefore, coaches need to understand that distance covered in all speed zones is only part of the picture and players particularly in the front and second rows might not run as far but will experience fatigue as a result of static work (scrums and mauls). In the future, it is suggested that the monitoring of in match heart rate will help build a better overall picture in conjunction with GPS and AS of the internal and external load players experience.

A further study by Tee and colleagues [67] reported that forward finishers (came off bench) completed more HSR and had a higher acceleration frequency than whole game players and starters (subbed off). The authors propose that this is a result of the finishers knowing how long is left in the match and therefore due, to the shorter durations, they are able to exert themselves more [67]. However, both these studies only investigated a single team and had a small sample size. Therefore, more research with different teams playing in different competitions is required to add to the literature.

Although further research is required, these studies could provide valuable insights which could be of interest to coaches who, using this information, may be able to better time tactical substitutions to influence the outcome of the matches. Understanding that forwards fatigue more quickly and finishers complete more HSR may also influence the breakdown of the substitutes’ bench. Coaches may in certain games (based on the game plan) decide to reduce the numbers of backs on the bench (for example a six, two split of forwards and backs) in order to bring on an almost entirely new set of forwards who may be able to influence the game when competing against tiring opposition players.

### Impacts and Collisions During Match Play

The results of the eleven studies [23, 25, 26, 30, 33, 46, 50, 53, 58, 67, 68] which included collisions and impacts recorded from accelerometers imbedded within GPS units are included in Table 5. A number of studies utilising GPS and AS have reported that forwards were involved in more impacts and collisions compared to backs [26, 30, 33, 55, 58]. In contrast, Tee et al. [68] reported no significant differences in the total amount of impacts between backs and forwards (> 5 G min^−1^ and > 8 G min^−1^) in professional RU players. When assessing the magnitude and frequency of impacts, Grainger and colleagues [53] reported backs had more low-intensity impacts (3.01–5 G) than forwards; however, forwards had more high-intensity impacts (> 13 G). In contrast to their hypothesis, no differences were found between backs and forwards for both absolute and relative impacts > 9.01 G [53].. The authors also proposed that sensor impacts during match play were likely to be as a result of collisions and also impacts due to decelerations, landing and change of direction [53]. This led them to conclude that values reported from the AS imbedded in GPS units should be treated with caution as different tasks will result in differing levels of match fatigue which will influence future planning [53]. Currently, the overall picture is also complicated by the use of various classifications used for assessing impacts and collisions across different studies.

**Table 5:** Summary of impacts and collisions monitored by GPS units

Study	Participants	Forwards	Backs
Coughlan et al. [23]	2 players (1 back and 1 forward) from an international team.	**Impacts 5–5.99 G** 472 **Impacts 6–6.49 G** 132 **Impacts 6.5–6.99 G** 66 **Impacts 7–7.99 G** 103 **Impacts 8–9.99 G** 53 **Impacts > 10 G** 10	**Impacts 5–5.99 G** 353 **Impacts 6–6.49 G** 65 **Impacts 6.5–6.99 G** 48 **Impacts 7–7.99 G** 54 **Impacts 8–9.99 G** 40 **Impacts > 10 G** 13
Grainger et al. [53]	38 professional rugby union players from the English Premiership.	**Impacts 3.01–5 G** 1836 ± 604 **Impacts 5.01–7 G** 811 ± 243 **Impacts 7.01–9 G** 301 ± 133 **Impacts 9.01–11 G** 114 ± 79 **Impacts 11.01–13 G** 48 ± 41 **Impacts > 13 G** 66 ± 44	**Impacts 3.01–5 G** 2054 ± 546 **Impacts 5.01–7 G** 857 ± 297 **Impacts 7.01–9 G** 312 ± 154 **Impacts 9.01–11 G** 118 ± 79 **Impacts 11.01–13 G** 47 ± 38 **Impacts > 13 G** 59 ± 40
Jones et al. [30]	36 professional rugby union players.	**Total Contacts**—**measured using Catapults tackle detection metric** 31 ± 14	**Total Contacts**—**measured using Catapults tackle detection metric** 16 ± 7
Owen et al. [33]	33 professional rugby union players from a Super Rugby squad.	**1st half only** **Impacts 5–5.99 G** 218 ± 89 **Impacts 6–6.49 G** 66 ± 36 **Impacts 6.5–6.99 G** 45 ± 26 **Impacts 7–7.99 G** 53 ± 29 **Impacts 8–9.99 G** 25 ± 11 **Impacts > 10 G** 10	**1st half only** **Impacts 5–5.99 G** 176 ± 63 **Impacts 6–6.49 G** 51 ± 19 **Impacts 6.5–6.99 G** 35 ± 13 **Impacts 7–7.99 G** 43 ± 17 **Impacts 8–9.99 G** 22 ± 12 **Impacts > 10 G** 13
Pollard et al. [58]	22 players from an international rugby team.	**Collisions per min per position as detected by Statsport Software** 0.5 ± 0.1	**Collisions per min per position as detected by Statsport Software** 0.3 ± 0.1
Reardon et al. [34]	36 professional rugby union players from a Guinness Pro 12 team.	**Collisions 2.5 G threshold** Prop—34 ± 11 Hooker—33 ± 9 Second row—35 ± 11 Back row—44 ± 10	**Collision 3 G threshold** Scrum half—11 ± 6 Out-half—21 ± 7 Centre—20 ± 5 Wing—20 ± 5 Full back—21 ± 6
Suarez-Arrones et al. [26]	9 international rugby union players.	**Impacts 5–6 G** 501.6 ± 106 **Impacts 6–6.5 G** 341.3 ± 219 **Impacts 6.5–7 G** 161.6 ± 107 **Impacts 7–8 G** 143.1 ± 122 **Impacts 8–10 G** 66.6 ± 48 **Impacts > 10 G** 10.4 ± 5	**Impacts 5–6 G** 382 ± 126 **Impacts 6–6.5 G** 326 ± 173 **Impacts 6.5–7 G** 54.3 ± 28.9 **Impacts 7–8 G** 29.8 ± 9 **Impacts 8–10 G** 35.2 ± 26 **Impacts > 10 G** 6.3 ± 4
Tee et al. [50]	19 professional rugby union players from a South African rugby team.	**Total Impacts > 5 G min**^−**1**^ **1st half** 8.7 ± 2.4 **Total Impacts > 8 G min**^−**1**^ **1st half** 0.8 ± 0.3 **Total Impacts > 5 G min**^−**1**^ **2nd half** 7.9 ± 3.2 **Total Impacts > 8 G min**^−**1**^ **2nd half** 0.7 ± 0.3	**Total Impacts > 5 G min**^−**1**^ **1st half** 10.0 ± 3.5 **Total Impacts > 8 G min**^−**1**^ **1st half** 1.1 ± 0.3 **Total Impacts > 5 G min**^−**1**^ **2nd half** 9.0 ± 3.0 **Total Impacts > 8 G min**^−**1**^ **2nd half** 1.1 ± 0.4
Tee et al. [67]	19 professional rugby union players from a South African rugby team.	**Impacts > 5 G min**^−**1**^ 8.3 ± 2.7	**Impacts > 5 G min**^−**1**^ 9.5 ± 3.1
Tee et al. [68]	19 professional rugby union players from a South African rugby team.	**Total Impacts > 5 G min**^−**1**^ 10.0 ± 3 **Total Impacts > 8 G min**^−**1**^ 0.9 ± 0.4	**Total Impacts > 5 G min**^−**1**^ 9.5 ± 3.2 **Total Impacts > 8 G min**^−**1**^ 1.1 ± 0.4
Venter [25]	17 academy rugby union players from a South African team.	**Not all positions reported** **Impacts > 5 G** Back row forwards—683.4 ± 295.04 **Impacts > 10 G** Front row forwards—8 ± 4.58	**Not all positions reported** **Impacts > 5 G** Outside backs—474.33 ± 81.92 **Impacts > 10 G** Inside backs—12.16 ± 3.18

Macleod and colleagues [55] reported that forwards were involved in significantly more collisions than backs and a greater number of collisions per minute. When considering impact velocities, MacLeod et al. [55] reported that the majority of backs entered collisions at significantly higher velocities than the forwards. The authors propose that this is the result of forwards standing closer to the break down and therefore not being able to reach higher velocities before a collision occurs whereas the backs have greater space to generate higher velocities prior to a collision [55]. Owen et al. [33] reported that the forwards experienced more low-intensity impacts due to collisions occurring at low speeds. This led the authors to conclude that the ratio of high-intensity to low-intensity impacts was greater for backs [33]. However, the sheer number of impacts for forwards resulted in a greater absolute total of high-intensity impacts [33]. The authors suggest that recovery strategies may differ between backs and forwards with forwards needing greater recovery periods as a result of the greater aggregated body demands [33]. It stands to reason that due to their roles, forwards should be conditioned during training to ensure they are ready for the impact demands that are placed on them during match play [33]. The current authors propose that a careful balancing act is required between the need to ensure that forwards, in particular, are conditioned to be able to deal with repeated impacts but also the need to make sure they are fresh to play in matches while reducing the risk of training-based injuries.

While some studies have reported that GPS units with integrated AS were able to accurately and reliably record impacts [55] and collisions, others have not [46]. Despite the improvements in technology, there are still concerns about the ability of accelerometers to assess impacts which occur with very little horizontal displacement (rucks, mauls and tackles) [5]. As a result, it has been suggested that the true demands may still be being underestimated [5]. This then may result in clubs not using these metrics as was observed by West and colleagues [5] who reported that only two out of the twelve clubs in the English Premiership actually used the contact metrics available from AS contained within GPS units. Although advances are being made in this area, there is still some way to go before teams fully embrace these units to monitor impacts and collisions. Of some promise however is the recent work by Chambers et al. [64, 65] who used AS data to custom build algorithms which we able to automatically detect rucks, tackles [65] and scrums [64] which could potentially save a lot of time by reducing the need for someone to code these manually. While this is an exciting development, it does rely on having someone at the team who is able to write these algorithms and correctly interpret what they are showing. Therefore, it is suggested more work is needed in this area to ensure its validity and reliability before collision and impact data become more widely used.

### Peak Periods of Play

The results of studies which investigated the peak periods of play in senior rugby, are summarised in Table 6. In a study which investigated the use of rolling averages to identify the maximal mean demands (ranging from 1 to 10 min) for two international teams, the authors reported that the peak intensities achieved during competition were considerably higher than those previously reported using whole-period averages [6]. Delaney et al. [6] also investigated differences in positional groups identifying that outside backs and half backs covered greater relative distances compared to the tight five across all rolling average periods. In addition, it was found that loose forwards covered greater relative distance compared to the tight five and therefore the authors suggest these positions should be trained separately if coaches are prescribing training sessions based on running intensity [6]. In a further study which compared and contrasted the use of fixed time epochs vs rolling averages, Cunningham and colleagues [3] reported that the fixed time epoch method underestimated both the maximum distance covered and HSR regardless of the epoch length for both the team overall and when analysed by position group. In conclusion, the authors propose that when analysing GPS data, teams should employ the rolling method to allow for the accurate prescription of training loads and ensure that positional differences are acknowledged in training prescription. It should be noted however that neither the Delaney et al. [6] nor Cunningham et al. [3] studies included any information on impacts that occurred. This is an area which warrants further attention when designing sessions that aim to replicate the demands, as forwards and backs have differing roles during these periods.

**Table 6:** Rugby Union peak movement characteristics

Study	Participants	Average duration (s) or time epoch (s)	Relative distance (m.min^**−1**^)	High-speed running (m or m.min^**−1**^)
Cunningham et al. [3]	119 elite professional players from three different international performance squads.	60 rolling average and fixed	Rolling Average Fwds 156.5 ± 19.0 m.min^−1^ Backs 177.4 ± 20.6 m.min^−1^ Fixed Fwds 139.0 ± 38.2 m.min^−1^ Backs 160.1 ± 21.1 m.min^−1^	Rolling Average Fwds 42.5 ± 20.6 m.min^−1^ Backs 69.9 ± 21.8 m.min^−1^ Fixed Fwds 38.2 ± 17.5 m.min^−1^ Backs 63.2 ± 20.2 m.min^−1^
		120 rolling average and fixed	Rolling Average Fwds 123.7 ± 15.4 m.min^−1^ Backs 140.1 ± 16.3 m.min^−1^ Fixed Fwds 111.1 ± 22 m.min^−1^ Backs 126.9 ± 16.7 m.min^−1^	Rolling Average Fwds 24.9 ± 15.0 m.min^−1^ Backs 42.6 ± 15.7 m.min^−1^ Fixed Fwds 22.0 ± 13.3 m.min^−1^ Backs 36.9 ± 14.0 m.min^−1^
		180 rolling average and fixed	Rolling Average Fwds 109.2 ± 14.6 m.min^−1^ Backs 123.4 ± 15.4 m.min^−1^ Fixed Fwds 96.9. ± 16.1 m.min^−1^ Backs 110.6 ± 15.0 m.min^−1^	Rolling Average Fwds 18.9 ± 14.0 m.min^−1^ Backs 32.7 ± 14.0 m.min^−1^ Fixed Fwds 16.1 ± 13.3 m.min^−1^ Backs 27.4 ± 12.0 m.min^−1^
		240 rolling average and fixed	Rolling Average Fwds 101.0 ± 12.9 m.min^−1^ Backs 114.2 ± 14.4 m.min^−1^ Fixed Fwds 90.6 ± 13.2 m.min^−1^ Backs 102.0 ± 13.4 m.min^−1^	Rolling Average Fwds 15.4 ± 12.1 m.min^−1^ Backs 27.6 ± 12.2 m.min^−1^ Fixed Fwds 13.2 ± 10.0 m.min^−1^ Backs 22.8 ± 10.1 m.min^−1^
		300 rolling average and fixed	Rolling Average Fwds 95.4 ± 12.2 m.min^−1^ Backs 107.5 ± 13.3 m.min^−1^ Fixed Fwds 85.7 ± 10.9 m.min^−1^ Backs 96.5 ± 13.6 m.min^−1^	Rolling Average Fwds 13.1 ± 10.2 m.min^−1^ Backs 24.0 ± 10.8 m.min^−1^ Fixed Fwds 10.9 ± 7.3 m.min^−1^ Backs 20.0 ± 8.5 m.min^−1^
Delaney et al. [6]	67 players from two international rugby union teams.	60 rolling average	Tight Fwds 154 ± 210 m.min^−1^ Loose Fwds 169 ± 230 m.min^−1^ Half backs 184 ± 280 m.min^−1^ OB 175 ± 220 m.min^−1^	
		120 rolling average	Tight Fwds 122 ± 170 m.min^−1^ Loose Fwds 135 ± 160 m.min^−1^ Half backs 147 ± 210 m.min^−1^ OB 137 ± 160 m.min^−1^	
		180 rolling average	Tight Fwds 105 ± 150 m.min^−1^ Loose Fwds 117 ± 140 m.min^−1^ Half backs 126 ± 180 m.min^−1^ OB 119 ± 130 m.min^−1^	
		240 rolling average	Tight Fwds 96 ± 130 m.min^−1^ Loose Fwds 108 ± 130 m.min^−1^ Half backs 117 ± 160 m.min^−1^ OB 109 ± 120 m.min^−1^	
		300 rolling average	Tight Fwds 91 ± 120 m.min^−1^ Loose Fwds 102 ± 110 m.min^−1^ Half backs 108 ± 150 m.min^−1^ OB 103 ± 100 m.min^−1^	
		360 rolling average	Tight Fwds 88 ± 120 m.min^−1^ Loose Fwds 97 ± 110 m.min^−1^ Half backs 103 ± 140 m.min^−1^ OB 98 ± 100 m.min^−1^	
		420 rolling average	Tight Fwds 85 ± 110 m.min^−1^ Loose Fwds 94 ± 110 m.min^−1^ Half backs 100 ± 130 m.min^−1^ OB 95 ± 100 m.min^−1^	
		480 rolling average	Tight Fwds 82 ± 110 m.min^−1^ Loose Fwds 92 ± 110 m.min^−1^ Half backs 97 ± 120 m.min^−1^ OB 93 ± 90 m.min^−1^	
		540 rolling average	Tight Fwds 81 ± 110 m.min^−1^ Loose Fwds 90 ± 110 m.min^−1^ Half backs 95 ± 120 m.min^−1^ OB 90 ± 90 m.min^−1^	
		600 rolling average	Tight Fwds 79 ± 110 m.min^−1^ Loose Fwds 88 ± 110 m.min^−1^ Half backs 93 ± 120 m.min^−1^ OB 89 ± 90 m.min^−1^	
Flanagan et al. [44]	42 rugby union U20 international players across two teams.	5-min rolling period	Prop—58 ± 3.8 m.min^−1^ Hooker—58 ± 8 m.min^−1^ SR—56 ± 7 m.min^−1^ BR—60 ± 9 m.min^−1^ SH—71 ± 4 m.min^−1^ FH—69 ± 4 m.min^−1^ Centre—66 ± 7 m.min^−1^ B3—67 ± 6 m.min^−1^	Prop—42 ± 42 m Hooker—88 ± 88 m SR—55 ± 66 m BR—153 ± 65 m SH—191 ± 80 m FH—123 ± 29 m Centre—363 ± 120 m B3—514 ± 153 m
Pollard et al. [58]	22 players from an international rugby team.	30–60 ball in play	Fwds 106.9 ± 5.6 m.min^−1^ Backs 109.6 ± 11.4 m.min^−1^	Fwds 10.9 ± 4.7 m.min^−1^ Backs 20.3 ± 5.7 m.min^−1^
		61–90 ball in play	Fwds 104.6 ± 6.1 m.min^−1^ Backs 115.1 ± 11.4 m.min^−1^	Fwds 7.0 ± 4.1 m.min^−1^ Backs 18.9 ± 5.1 m.min^−1^
		> 90 ball in play	Fwds 105.0 ± 8.5 m.min^−1^ Backs 110.9 ± 9.5 m.min^−1^	Fwds 5.8 ± 2.7 m.min^−1^ Backs 15.6 ± 5.8 m.min^−1^
Read et al. [61]	202 rugby union players across 7 regional academies in England.	15 rolling average	FR 245 ± 32 m.min^−1^ SR 264 ± 29 m.min^−1^ BR 280 ± 36 m.min^−1^ SH 298 ± 44 m.min^−1^ IB 297 ± 33 m.min^−1^ OB 299 ± 42 m.min^−1^	
		30 rolling average	FR 193 ± 21 m.min^−1^ SR 207 ± 19 m.min^−1^ BR 217 ± 23 m.min^−1^ SH 233 ± 25 m.min^−1^ IB 233 ± 23 m.min^−1^ OB 244 ± 30 m.min^−1^	
		60 rolling average	FR 154 ± 17 m.min^−1^ SR 165 ± 12 m.min^−1^ BR 168 ± 19 m.min^−1^ SH 185 ± 20 m.min^−1^ IB 172 ± 19 m.min^−1^ OB 170 ± 22 m.min^−1^	
		120 rolling average	FR 121 ± 16 m.min^−1^ SR 130 ± 12 m.min^−1^ BR 132 ± 15 m.min^−1^ SH 146 ± 19 m.min^−1^ IB 135 ± 16 m.min^−1^ OB 133 ± 17 m.min^−1^	
		150 rolling average	FR 112 ± 15 m.min^−1^ SR 121 ± 13 m.min^−1^ BR 123 ± 14 m.min^−1^ SH 138 ± 18 m.min^−1^ IB 128 ± 16 m.min^−1^ OB 124 ± 15 m.min^−1^	
		180 rolling average	FR 106 ± 14 m.min^−1^ SR 115 ± 14 m.min^−1^ BR 116 ± 14 m.min^−1^ SH 132 ± 17 m.min^−1^ IB 120 ± 14 m.min^−1^ OB 118 ± 15 m.min^−1^	
		240 rolling average	FR 99 ± 14 m.min^−1^ SR 106 ± 12 m.min^−1^ BR 108 ± 14 m.min^−1^ SH 122 ± 15 m.min^−1^ IB 112 ± 13 m.min^−1^ OB 111 ± 14 m.min^−1^	
		300 rolling average	FR 93 ± 14 m.min^−1^ SR 100 ± 12 m.min^−1^ BR 102 ± 14 m.min^−1^ SH 116 ± 14 m.min^−1^ IB 106 ± 12 m.min^−1^ OB 104 ± 14 m.min^−1^	
		600 rolling average	FR 80 ± 12 m.min^−1^ SR 87 ± 9 m.min^−1^ BR 88 ± 11 m.min^−1^ SH 97 ± 13 m.min^−1^ IB 92 ± 10 m.min^−1^ OB 89 ± 11 m.min^−1^	
Read et al. [60]	59 rugby union academy players from England.	0–15	Attack Fwds—103.3 ± 62.2 m.min^−1^ Attack Backs—102.0 ± 64.2 m.min^−1^ Defence Fwds—109.4 ± 67.1 m.min^−1^ Defence Backs—106.5 ± 68.6 m.min^−1^	
		16–30	Attack Fwds—115.9 ± 44.8 m.min^−1^ Attack Backs—118.3 ± 50.4 m.min^−1^ Defence Fwds—118.4 ± 52.5 m.min^−1^ Defence Backs—110.5 ± 54.5 m.min^−1^	
		31–45	Attack Fwds—118.3 ± 35.6 m.min^−1^ Attack Backs—124.2 ± 39.2 m.min^−1^ Defence Fwds—117.4 ± 35.5 m.min^−1^ Defence Backs—113.2 ± 41.1 m.min^−1^	
		46–60	Attack Fwds—116.9 ± 28.6 m.min^−1^ Attack Backs—121.9 ± 33.4 m.min^−1^ Defence Fwds—112.6 ± 30.9 m.min^−1^ Defence Backs—106.7 ± 34.3 m.min^−1^	
		> 60	Attack Fwds—112.7 ± 23.3 m.min^−1^ Attack Backs—118.7 ± 29.8 m.min^−1^ Defence Fwds—108.4 ± 20.9 m.min^−1^ Defence Backs—102.0 ± 28.2 m.min^−1^	
Reardon et al. [47]	39 professional rugby union players from a Pro12 team.	Tight Fwds 161 BR Fwds 152 IB 154 OB 155	Tight Fwds 109 m.min^−1^ BR Fwds 111 m.min^−1^ IB 123 m.min^−1^ OB 124 m.min^−1^	Tight Fwds 4.9 m.min^−1^ BR Fwds 6.0 m.min^−1^ IB 8.1 m.min^−1^ OB 14.1 m.min^−1^
Tierney et al. [51]	43 professional rugby union players from a Pro 12 team.	Attacking 22 entries	Prop—57.7 m.min^−1^ (44.1–63.4) Hooker—63.2 m.min^−1^ (49.6–76.9) SR—51.5 m.min^−1^ (41.5–61.4) BR—54.5 m.min^−1^ (46.6–62.3) SH—76.8 m.min^−1^ (62.9–90.7) FH—62.2 m.min^−1^ (49.7–74.7) Centre—61.2 m.min^−1^ (49.2–73.3) B3—58.8 m.min^−1^ (51.3–66.2)	Prop—2.3 m.min^−1^ (− 1.1–5.8) Hooker—6.5 m.min^−1^ (1.6–11.4) SR—2.3 m.min^−1^ (− 1.3–5.9) BR 2.4 m.min^−1^ (− 0.4–5.2) SH—11.5 m.min^−1^ (6.5–16.4) FH—7.0 m.min^−1^ (2.5–11.5) Centre—8.1 m.min^−1^ (3.8–12.4) B3—10.2 m.min^−1^ (7.5–12.8)

*Fwds* forwards, *FR* front row, *TF* tight forwards, *SR* second row, *BR* back row, *SH* scrum half, *FH* fly half, *IB* inside back, *OB* outside back, *B3* back 3

Pollard and colleagues [58] investigated the peak demands of international rugby union via GPS with the results reported as mean BiP, maximum BiP and whole match outputs. The authors reported whole match metrics were significantly lower than all BiP metrics adding further evidence to the suggestion that merely analysing whole match GPS metrics does not give an accurate representation of locomotive intensity. Using this method, the authors reported significantly higher mean and maximum BiP and HSR for backs versus forwards; however, forwards had a significantly higher number of collisions [58] in agreement with previous research [10, 69]. The authors also reported that during all maximum BiP periods GPS metrics decreased over time, with the highest outputs observed in periods lasting 30–60 s and the lowest in periods over 90 s which is in broad agreement with the rolling average study conducted by Delaney et al [6].

In club rugby, Reardon et al. [47] investigated the demands of the single longest period of ball in play over the course of a season and termed this the worst-case scenario (WCS). The authors found that the majority of work recorded during the most intense period of play was carried out at low-intensity with intermittent bouts of high-intensity running [47]. It was also reported that the average intensity of this period was far greater than the previously reported average game demands (117 m.min^−1^ vs 68 m.min^−1^) [47]. The differences between backs and forwards were also pronounced during these periods with backs covering greater total distances (318 m vs 289 m), more high-speed running (11.1 m.min^−1^ vs 5.5 m.min^−1^) and achieving higher maximum velocities [47]. This study provides further evidence of the need to ensure that where possible training reflects positional differences, and the different locomotor demands associated with these positions.

When investigating the demands of age-grade international RU, Flanagan et al. [44] reported that the relative distance covered during the peak 5-min period in matches ranged between 77 and 100 m.min^−1^ which was 33 to 48% greater than the mean relative distance covered during the game. While using the rolling average method, Read et al. [61] investigated maximum running intensities during English academy RU matches. The key findings from this study were that the running intensities of U18 front row players are different from those of second row and back row players and scrum halves were different from both inside and outside backs (apart from 15 s and 30 s time epochs) [61]. The authors propose that the data from this study could be used as a reference for academy players when designing drills to replicate the most intense periods of play [61]. In addition, they suggest that due to the differences observed coaches should make special considerations when designing sessions for both front row players and scrum halves [61].

Read and colleagues [60] investigated the characteristics of attacking, defending and ball in play and out of play, in academy forwards and backs during match play. The mean relative distance covered during attack and defence was reported to range between 109.0 and 114.6 m.min^−1^ [60]. It was also reported that PlayerLoad (PL·min^−1^, Catapult) was *almost certainly* greater in forwards when both attacking and defending; this was attributed to forwards completing more running, carries, tackles and rucks [60]. The authors suggest that a novel finding of this study is that academy backs cover an *almost certainly* greater distance than forwards when the ball is out of play (e.g. lineouts and scrums getting set) with this proposed to be the result of backs having to reposition themselves on the field while waiting for play to resume [60]. In terms of position-specific phase demands, *likely trivial* differences between PL and relative distance covered during both attacking and defending were reported suggesting that both attacking and defending in academy forwards can be prepared for in a similar way [60]. However, academy backs were found to have *likely* greater differences in relative distance covered and PL in attack compared to defence indicating backs are more involved in attacking plays in comparison to defence [60]. It is acknowledged, however, that this analysis may underestimate the most intense periods of play as this could involve being on the attack losing the ball and having to switch to defending [60]. A further study investigating peak periods by Tierney et al. [51] reported that forwards achieved greater HSR intensity (3.6 m.min^−1^ vs 1.8 m.min^−1^) in successful visits to the 22 compared to unsuccessful visits. This the authors propose is a result of the forwards working harder to be in position, to support the next phase of play [51]. Surprisingly, backs were reported to have significantly lower running intensity, HSR and very-high-intensity running during successful attacking 22 entries compared to unsuccessful entries [51]. The authors suggest that this is the result of backs having to work harder during unsuccessful 22 entries to account for the lower work rate of forwards [51]. Based on the results of this study, it was suggested that forwards should be conditioned to be able to repeatedly achieve greater HSR efforts in attacking 22 scenarios to increase the likelihood of a try being scored [51].

In summary, it is suggested that information recorded by the GPS unit during the most intense periods of play could be utilised by coaches to design drills that replicate or even exceed the locomotor demands imposed during match play. Based on the results of studies in this review, where possible it would appear that the rolling average method is preferable to the fixed average method. When designing the drills, it is also proposed that a one size fits all model is inappropriate and therefore training should where possible take into account positional differences. It is important to note, however, that the majority of the periods described only included locomotor activities, and therefore, without knowing what else is going on during these periods (i.e. mauling, tackling, rucking), it is hard to say whether these periods represent the most intense periods players and in particular forwards truly encounter during matches.

### Training Sessions

A total of three senior [43, 52, 62] and four age-grade studies [24, 45, 56, 57] reported training loads utilising GPS (Table 7). Training sessions are essential in RU in order to allow the players to tolerate the demands of the competition, express themselves on the pitch, make decisions, execute skills under fatigue, recover quickly and reduce the chances of them getting injured [24, 56, 57]. It has previously been proposed that closely simulating game demands during training will help optimally prepare players to perform on match day [40]. In studies using GPS to investigate the movement patterns of training sessions and matches, differences between the two have been identified [40, 52]. Tee et al. [40] observed that players walked more during matches than during training (ES = medium to large), and the authors suggested that this was the result of the intermittent nature of RU where regular stoppages result in players walking to the next phase of play (i.e. from penalty kick to the resultant lineout). In a further study, Campbell et al. [52] reported that outside backs, loose forwards and front row forwards covered greater total distance and loose forwards and front row players covered greater relative distances in matches compared to training. The results of Campbell et al.’s [52] study suggest that in some positions, training may not optimally prepare players to perform on match day. However, the current authors proposed that these results may be due to there being a much greater focus on static unit work such as scrums and lineouts for the forwards in particular during training sessions. Therefore, GPS data alone may not be representative of the demands of training, and as a result, an internal measure of load such as heart rate may also be valuable.

**Table 7:** Summary of GPS use in age-grade and senior training sessions

Study	Participants	Forwards	Backs
Campbell et al. [52]	32 club rugby union players.	**Mean total session distance (m)**—**Front row** 4074 ± 974 m **Mean total session distance (m)**—**Locks** 4698 ± 1120 m **Mean total session distance (m)**—**Loose forwards** 4173 ± 1003 m **Mean total session HSR (> 12.5 ms**^−**1**^**) distance (m)**—**Front row** 91.1 ± 80.2 m **Mean total session HSR (> 12.5 ms**^−**1**^**) distance (m)**—**Locks** 211 ± 208 m **Mean total session HSR (> 12.5 ms**^−**1**^**) distance (m)**—**Loose forwards** 129 ± 156 m	**Mean total session distance (m)**—**Halves** 5259 ± 1345 m **Mean total session distance (m)**—**Centres** 5217 ± 1208 m **Mean total session distance (m)**—**Outside backs** 4978 ± 1203 m **Mean total session HSR (> 12.5 ms**^−**1**^**) distance (m)**—**Halves** 227 ± 230 m **Mean total session HSR (> 12.5 ms**^−**1**^**) distance (m)**—**Centres** 307 ± 173 m **Mean total session HSR (> 12.5 ms**^−**1**^**) distance (m)**—**Outside backs** 320 ± 202 m
Dubios et al. [43]	8 professional rugby union players (all backs) from the D2 Championship in France.		**Mean total weekly distance (m)** 19316 ± 2923 m **Mean weekly HSR (> 8.1 ms**^−**1**^**) distance (m)** 3996 ± 701 m
Hartwig et al. [24]	118 rugby union players aged between 14 and 18 years.	**Percentage time stationary (0–0.6 ms**^−**1**^**)**—**games Vs training** 44.5 ± 4.3% Vs 44.9 ± 10.4% **Percentage time walking (0.6–4.3 ms**^−**1**^**)**—**games Vs training** 35.3 ± 4.2% Vs 45.0 ± 8.8% **Percentage time jogging (4.3–7.5 ms**^−**1**^**)**—**games Vs training** 14.5 ± 2.7% Vs 8.6 ± 3.1% **Percentage time striding (7.5–13.1 ms**^−**1**^**)**—**games Vs training** 3.6 (3.5) Vs 1.1 (1.1) median and interquartile range **Percentage time sprinting (> 13.1 ms**^−**1**^**)**—**games Vs training** 0.9% (2.1) Vs 0.001% (0.1) median and interquartile range **Sprints per hour of play**—**games Vs training** 17.6 ± 38.9 Vs 0.7 ± 2.5 **Sprint distance per hour of play (m)**—**games Vs training** 220 ± 552 m Vs 6.4 ± 30.8 m **Sprint distance (m)**—**games Vs training** 12.3 ± 5.1 Vs 11.5 ± 8.4 m	**Percentage time stationary (0–0.6 ms**^−**1**^**)**—**games Vs training** 32.7 ± 7.3% Vs 40.7 ± 10.3% **Percentage time walking (0.6–4.3 ms**^−**1**^**)**—**games Vs training** 48.8 ± 7.6% Vs 48.1 ± 7.7% **Percentage time jogging (4.3–7.5 ms**^−**1**^**)**—**games Vs training** 13.6 ± 2.5% Vs 9.1 ± 4.1% **Percentage time striding (7.5–13.1 ms**^−**1**^**)**—**games Vs training** 3.1 (1.8%) Vs 1.8 (1.5) median and interquartile range **Percentage time sprinting (> 13.1 ms**^−**1**^**)**—**games Vs training** 1.3 (0.8) Vs 0.1 (0.4) median and interquartile range **Sprints per hour of play**—**games Vs training** 22 ± 11.2 Vs 2.1 ± 5.9 **Sprint distance per hour of play (m)**—**games Vs training** 346 ± 231 m Vs 20.8 ± 96.6 m **Sprint distance (m)**—**games Vs training** 13.6 ± 4.8 m Vs 11.3 ± 10.6 m
Phibbs et al. [45]	170 adolescent rugby union players (U16 and U18).	No differentiation between positions (under 16 s) **Mean session total distance (m)**—**School** 2672 ± 456 m **Mean session total distance (m)**—**Club** 3619 ± 664 m **Mean session total distance (m)**—**Academy** 2903 ± 434 m **Mean session total HSR (7.5–13.1 ms**^−**1**^**) distance (m)**—**School** 751 ± 242 m **Mean session total HSR (7.5–13.1 ms**^−**1**^**) distance (m)**—**Club** 955 ± 256 m **Mean total session HSR (7.5–13.1 ms**^−**1**^**) distance (m)**—**Academy** 590 ± 219 m	No differentiation between positions (under 18 s) **Mean total distance (m)**—**School** 2925 ± 467 m **Mean session total distance (m)**—**Club** 3845 ± 577 m **Mean session total distance (m)**—**Academy** 4176 ± 433 m **Mean session total HSR (7.5–13.1 ms**^−**1**^**) distance (m)**—**School** 678 ± 179 m **Mean session total HSR (7.5–13.1 ms**^−**1**^**) distance (m)**—**Club** 597 ± 246 m **Mean session total HSR (7.5–13.1 ms**^−**1**^**) distance (m)**—**Academy** 1270 ± 288 m
Phibbs et al. [57]	20 adolescent academy rugby union players.	**Mean weekly total distance (m)** 10195 ± 2242 m **Mean weekly HSR (> 61% Vmax) distance (m)** 482 ± 174 m **Mean weekly VHSR (> 90% Vmax) distance (m)** 5 ± 8 m	**Mean weekly total distance (m)** 13063 ± 3933 m **Mean weekly HSR (> 61% Vmax) distance (m)** 807 ± 387 m **Mean weekly VHSR (> 90% Vmax) distance (m)** 34 ± 51 m
Phibbs et al. [56]	61 adolescent schoolboy and academy rugby union players.	**Mean session total distance (m)**—**School** 3433 ± 300 m **Mean session total distance (m)**—**Academy** 4031 ± 755 m **Mean session HSR (> 61% maximal sprint speed)**—**School** 276 ± 71 m **Mean session HSR (> 61% maximal sprint speed)**—**Academy** 252 ± 120 m	**Mean session total distance (m)**—**School** 3821 ± 386 m **Mean session total distance (m)**—**Academy** 4678 ± 356 m **Mean session HSR (> 61% maximal sprint speed)**—**School** 275 ± 105 m **Mean session HSR (> 61% maximal sprint speed)**—**Academy** 345 ± 160 m
Weaving et al. [62]	21 professional rugby union players.	**No positions identified in the results** **Mean total session distance (m)** 3096 ± 675 m **Mean total session HSR (> 61% maximal sprint speed)** 127 ± 202 m	

When investigating specific types of training sessions using GPS, Tee et al. [40] reported that overall high-intensity interval training was the training activity most specific to match play. Game-based training was found to be specific to match play requirements in relation to speed and acceleration variables [40] in agreement with previous research that proposes it as an appropriate training method for RU [70]. However, it should be noted that when compared to requirements for specific players in some positions, it was not always able to replicate match play intensity. Therefore, if a team were just to use a game-based conditioning approach not every player in every position may get the optimal training stimulus. This would potentially reduce their ability to perform on match day or increase their risk of injury as they are performing at intensities at which they are inefficient or unaccustomed to. It should also be noted, as with many other GPS studies, contacts were not reported and therefore these may still be a missing piece of the jigsaw puzzle when determining the internal and external load of both training activities and match play. The authors of this study also reported that no training activities managed to replicate the maximum speed requirements of outside backs during back play [40]. This suggests that outside backs need to be regularly exposed to maximum speed training in order to prepare them for the demands of match play [40]. Indeed, Malone et al. [71] reported that Gaelic footballers who produced ≥ 95% of their maximum velocity were at a reduced risk of soft tissue injury. Therefore, the authors suggest that exposure to maximal velocity sprinting during training may offer protection against subsequent soft tissue injury [71].

In age-grade rugby similar to senior rugby, it has been reported that there is a disparity between what is observed in match play compared to what the players do in training [24, 56]. Hartwig et al. [24] reported that age-grade players (aged 14–18) covered greater distances and completed more sprints during matches compared to training. Therefore, it is proposed that as with the senior players coaches should be ensuring that younger players receive exposure to maximum velocity running during training to reduce the risk of injury and also to potentially enhance performance. In a study which compared session training loads between different ages and playing standards, it was reported that U18 academy players covered the greatest distance and completed the most HSR while the U16 schoolboys covered the lowest total distance [45]. This led the authors to conclude the demands of training increase with age and playing standard [45]. The authors also suggest that amateur clubs and schools may wish to adopt practices which result in similar intensities being achieved during training to those seen in the academy [45]. However, this may be hard to achieve as it is unlikely that schools and clubs will have access to GPS devices outside of training studies in order to properly monitor training intensity. There is also perhaps a danger that without adequate monitoring both volume and intensity could be too high resulting in an increased risk of injury and a reduction in performance.

A further study investigated training demands compared to match play in adolescent schoolboy and academy RU players. Phibbs et al. [56] reported schoolboy forwards were underprepared for low-intensity match activities and schoolboy backs were underprepared for match play movements. The authors observed that academy forwards were exposed to similar demands in training as in matches and the backs had similar values or indeed even exceeded match play values during training [56]. When examining the training data, it was found that both the schoolboy backs and forwards had similar movement demands placed on them which suggests a generic training approach [56]. The results of this study led the authors to conclude that training should be both position and playing standard specific in order to properly prepare adolescent players to perform optimally on match day and to reduce risk of injury [56].

In a study which investigated weekly training loads in academy players, it was reported that the backs had covered greater total distance, HSR and VHSR than the forwards which supports the idea that a position-specific approach to training occurs at the academy level [57]. The authors also reported a large within-subject variability in weekly training loads which could increase the risk of injury due to dips and spikes in a player’s workload [57]. As age-grade players may often represent a number of teams (school, club, county and academy), it is crucial that coaches and support staff at all levels are aware of the players’ weekly running loads. This will allow them to work together and plan appropriate training programmes which give the player the best chance of making the step up to higher honours while reducing the risk of them suffering an injury. In relation to the support staff relationship with the technical coaches planning skills sessions, it is suggested that the technical coach will require clear and concise information on training loads to help inform any decisions on session structure. A study by Weaving et al. [62] reported that practitioners could quantify training load using one of PlayerLoad, total distance or sRPE plus HSR distance. Therefore, relatively simple metrics obtained from GPS devices could be used to feedback information to the technical coaching staff.

When investigating the effects of including or excluding contact during training sessions in academy rugby, Roe et al. [49] reported that excluding contact training *almost certainly* increased running intensity (19.8 ± 5%) and total distance (27.5 ± 5.3%). As a consequence of excluding contact and therefore increasing running intensity and distance, the authors reported that the players had greater lower-body neuromuscular fatigue [49]. Therefore, when coaches are planning training sessions, it is suggested that they need to be aware of the consequences of including or excluding contact [49]. This information it is proposed could be useful to coaches when they are planning the training week in the lead up to match day.

### Limitations of the Review

One of the major limitations of this review is the inability to compare findings across studies to substantiate authors’ findings. This is due to a number of methodological and measurement concerns as highlighted by Ziv et al. [9] in their review of on field performance in RU. In this current review, data from GPS units were sampled at 5, 10 and 15 Hz thus making comparisons between studies difficult as different sampling rates have different levels of accuracy [9]. Previous research has identified that an increase in sampling rate from 1 to 5 Hz provided a more valid and reliable measurement of movement demands [17]. In addition, Varley et al. [72] reported that 10 Hz units were six times more reliable for measuring instantaneous velocity compared to 5 Hz units. These findings, therefore, suggest it is not appropriate to compare studies which utilised different GPS sample rates. In addition, the results captured from different GPS models should not be used interchangeably as different units have different bias when analysing locomotion [73]. Most of the studies used in this review also did not report the number of satellites connected and the horizontal dilution of position during data collection, which is also a concern.

### Future Directions

Moving forward, more work is required to allow GPS with imbedded AS to accurately monitor impacts and correctly identify events such as rucks, mauls and scrums in order for teams to have confidence that the device is accurately reporting what occurred. The authors also propose that future studies should also consider the context of the game when interpreting all variables.

## Conclusion

This review provides information on the current use of GPS and AS in both senior and age-grade RU. These allow support staff and coaches to assess and monitor performance both during training sessions and match play. Differences in running performance, collisions and impacts exist between forwards and backs based on their unique roles within the game. In future, it is also proposed that the context (game plan, opposition and level of competition) of the game needs much greater consideration when reporting GPS and AS data. This will allow coaches and support staff to analyse and interpret the data while also taking the big picture into account. This knowledge and information from GPS devices can be used to help prepare the players to meet the match-play demands and monitor training loads to help reduce injury risk.

## Supplementary Information

**Additional file 1: Table S1**. Methodological quality assessment.

## Data Availability

The data sets used and/or analysed during this study are available from the corresponding author on reasonable request.

## Abbreviations

BiP: Ball in play
ES: Effect size
GPS: Global positioning system
HSR: High-speed running
HMLD: High metabolic load distance
RU: Rugby union
RHIE: Repeated high-intensity efforts
RHILE: Repeated high-intensity locomotive efforts
VHSR: Very-high-speed running
WCS: Worst-case scenario
