# Supplementary material for: The Use of Global Positioning and Accelerometer Systems in Age-Grade and Senior Rugby Union: A Systematic Review
Source: Sports Med Open. 2021 Feb 22;7:15. doi: 10.1186/s40798-021-00305-x (PMC7900280; doi:10.1186/s40798-021-00305-x)
Supplement: Supplementary file 1 — Additional file 1: Table S1. Methodological quality assessment. [file 40798_2021_305_MOESM1_ESM.pdf]

The Use of Global Positioning and Accelerometer Systems in Age-Grade and Senior Rugby Union: A Systematic Review

Sports Medicine

Lee A. Bridgeman, PhD, [1] and Nicholas D. Gill, PhD, [2,3]

[1]Department of Sport and Exercise Science, Solent University, Southampton, England; [2]New Zealand Rugby Union, Wellington, New Zealand [3]Adams Centre for High Performance, Faculty of Health, Sport and Human Performance, University of Waikato, Tauranga, New Zealand

Corresponding authors contact details:

Lee Bridgeman

Email: lee.bridgeman@solent.ac.uk

Study	Criteria												Overall Score (12)
	1	2	3	4	5	6	7	8	9	10	11	12	
Beard et al. (63)	Y	Y	Y	Y	Y	Y	N	U	N	Y	Y	Y	9
Cahill (28)	Y	Y	Y	Y	Y	Y	N	U	N	Y	Y	Y	9
Campbell et al. (52)	Y	Y	Y	Y	Y	Y	Y	U	N	Y	Y	Y	10
Carling et al. (41)	Y	Y	Y	Y	Y	Y	ES	U	N	Y	Y	Y	9
Chambers et al. (64)	Y	Y	Y	Y	Y	Y	N/A	U	N	Y	Y	Y	9
Chambers et al. (65)	Y	Y	Y	Y	Y	Y	N/A	U	N	Y	Y	Y	9
Coughlan et al. (23)	Y	Y	Y	Y	Y	N	N	N	N	Y	N/A	N/A	6
Cousins et al. (66)	Y	Y	Y	Y	Y	Y	Y	U	N	Y	Y	Y	10
Cunningham et al. (36)	Y	Y	Y	Y	Y	Y	N	U	N	Y	Y	Y	9
Cunningham et al. (35)	Y	Y	Y	Y	Y	Y	N	U	N	Y	Y	Y	9
Cunningham et al. (3)	Y	Y	Y	Y	Y	Y	Y	U	N	Y	Y	Y	10
Delaney et al. (6)	Y	Y	Y	Y	Y	Y	ES	U	N	Y	Y	Y	9
Dubios et al. (4)	Y	Y	Y	Y	Y	Y	N	U	N	Y	Y	Y	9
Dubios et al. (43)	Y	Y	Y	Y	Y	Y	N	U	N	Y	Y	Y	9
Flanagan et al. (44)	Y	Y	Y	Y	Y	Y	ES	U	N	Y	Y	Y	9
Grainger et al. (53)	Y	Y	Y	Y	Y	Y	Y	U	N	Y	Y	Y	10
Hartwig et al. (24)	Y	Y	Y	Y	Y	Y	N	U	N	Y	Y	Y	9
Jones et al. (30)	Y	Y	Y	Y	Y	Y	N	U	N	Y	Y	Y	9
Jones et al. (31)	Y	Y	Y	Y	Y	Y	N	U	N	Y	Y	Y	9
Lacome et al. (54)	Y	Y	Y	Y	Y	Y	ES	U	N	Y	Y	Y	9
Lindsay et al. (32)	Y	Y	Y	Y	Y	Y	N	U	N	Y	Y	Y	9
MacLeod et al. (55)	Y	Y	Y	Y	Y	Y	Y	U	N	Y	Y	Y	10
Mclaren et al. (18)	Y	Y	Y	Y	Y	Y	N	U	N	Y	Y	Y	9
Owen et al. (33)	Y	Y	Y	Y	Y	Y	ES	U	N	Y	Y	Y	9
Phibbs et al. (45)	Y	Y	Y	Y	Y	Y	ES	U	N	Y	Y	Y	9
Phibbs et al. (56)	Y	Y	Y	Y	Y	Y	ES	U	N	Y	Y	Y	9
Phibbs et al. (57)	Y	Y	Y	Y	Y	Y	ES	U	N	Y	Y	Y	9
Pollard et al. (58)	Y	Y	Y	Y	Y	Y	Y	U	N	Y	Y	Y	10
Read et al. (14)	Y	Y	Y	Y	Y	Y	ES	U	N	Y	Y	Y	9
Read et al. (37)	Y	Y	Y	Y	Y	Y	ES	U	N	Y	Y	Y	9

Study	1	2	3	4	5	6	7	8	9	10	11	12	Overall Score (12)
Read et al. (59)	Y	Y	Y	Y	Y	Y	ES	U	N	Y	Y	Y	9
Read et al. (60)	Y	Y	Y	Y	Y	Y	ES	U	N	Y	Y	Y	9
Read et al. (61)	Y	Y	Y	Y	Y	Y	ES	U	N	Y	Y	Y	9
Reardon et al. (34)	Y	Y	Y	Y	Y	Y	Y	U	N	Y	Y	Y	10
Reardon et al. (47)	Y	Y	Y	Y	Y	Y	N	U	N	Y	Y	Y	9
Reardon et al. (46)	Y	Y	Y	Y	Y	Y	Y	U	N	Y	Y	Y	10
Reid et al. (29)	Y	Y	Y	Y	Y	Y	N/A	N	N	Y	N/A	N/A	7
Roe et al. (38)	Y	Y	Y	Y	Y	Y	ES	U	N	Y	Y	Y	9
Roe et al. (48)	Y	Y	Y	Y	Y	Y	N/A	U	N	Y	Y	Y	9
Roe et al. (49)	Y	Y	Y	Y	Y	Y	ES	U	N	Y	Y	Y	9
Suarez-Arrones et al. (26)	Y	Y	Y	Y	Y	Y	N	U	N	Y	Y	Y	9
Swaby et al. (39)	Y	Y	Y	Y	Y	Y	Y	U	N	Y	Y	Y	10
Tee et al. (68)	Y	Y	Y	Y	Y	Y	ES	U	N	Y	Y	Y	9
Tee et al. (50)	Y	Y	Y	Y	Y	Y	ES	U	N	Y	Y	Y	9
Tee et al. (40)	Y	Y	Y	Y	Y	Y	ES	U	N	Y	Y	Y	9
Tee et al. (67)	Y	Y	Y	Y	Y	Y	Y	U	N	Y	Y	Y	10
Tierney et al. (51)	Y	Y	Y	Y	Y	Y	Y	U	N	Y	Y	Y	10
Vaz et al. (27)	Y	Y	Y	Y	Y	Y	Y	U	N	Y	Y	Y	10
Vaz et al. (41)	Y	Y	Y	Y	Y	Y	Y	U	N	Y	Y	Y	10
Venter et al. (25)	Y	Y	Y	Y	Y	Y	N	U	N	Y	Y	Y	9
Weaving et al. (62)	Y	Y	Y	Y	Y	Y	N	U	N	Y	Y	Y	9

Table S1. Methodological quality assessment

Criteria - ES = effect sizes reported; U = Unable to determine, Y = Yes; N = No.

1. Hypothesis/aim clearly stated
2. Main outcomes described in introduction or method
3. Subject characteristics described
4. Intervention described
5. Main findings described
6. Estimate of random variability for main outcome
7. Actual probability value reported
8. Potential recruits representative of entire population
9. Participants representative of entire population from which they were recruited
10. Data dredging made clear
11. Statistical tests appropriate
12. Outcome measures valid and reliable
